# Sexual maturity and shape development in cranial appendages of extant ruminants

**DOI:** 10.1002/ece3.2512

**Published:** 2016-10-09

**Authors:** Zachary T. Calamari

**Affiliations:** ^1^ Richard Gilder Graduate School Division of Paleontology American Museum of Natural History New York NY USA

**Keywords:** comparative methods, cranial appendages, development, Ruminantia, sexual maturity, shape

## Abstract

Morphological disparity arises through changes in the ontogeny of structures; however, a major challenge of studying the effect of development on shape is the difficulty of collecting time series of data for large numbers of taxa. A proxy for developmental series proposed here is the age at sexual maturity, a developmental milestone potentially tied to the development of structures with documented use in intrasexual competition, such as cranial appendages in Artiodactyla. This study tested the hypothesis that ruminant cranial appendage shape and size correlate with onset of sexual maturity, predicting that late sexual maturity would correlate with larger, more complicated cranial appendages. Published data for cranial appendage shape and size in extant taxa were tested for correlations with sexual maturity using linear mixed‐effect models and phylogenetic generalized least‐squares analyses. Ancestral state reconstructions were used to assess correlated variables for developmental shifts indicative of heterochrony. These tests showed that phylogeny and body mass were the most common predictors of cranial appendage shape and sexual maturity was only significant as an interaction with body mass. Nevertheless, using developmental milestones as proxies for ontogeny may still be valuable in targeting future research to better understand the role of development in the evolution of disparate morphology when correlations exist between the milestone and shape.

## 1 Introduction

Understanding the roles of development in phenotypic evolution is a major goal in the study of evolution and development, or “evo‐devo” (Klingenberg, 2010). New structures and increased morphological disparity are known to arise through modification of the timing, rate, or direction of development (Zelditch, Sheets, & Fink, 2003; Zelditch, Swiderski, & Sheets, 2012), but development can be difficult to study in wild animals because exact ages are usually unknown and observational studies for many taxa would require an immense commitment of time and resources. Species that engage in intrasexual competition using external morphological characters (e.g., horns, claws, feathers) may present an opportunity to test the effect of the rate or duration of development on phenotypes without the need for multiple observations over time. Because these characters are largely associated with intrasexual competition over potential mates (Emlen, 2008), correlations between age at sexual maturity and the shape or size of these structures may indicate a link between readiness to compete and ability to do so effectively. In essence, animals may need to have their weapons or displays as close to adult form as possible by the time they reach sexual maturity to compete successfully for mating opportunities. Selection pressure to achieve an adultlike shape by sexual maturity could manifest as correlations between shape and the age of sexual maturity that suggest different developmental scenarios. A positive correlation may suggest that longer duration of development allows taxa to build larger or more ornate structures, while a negative correlation would suggest that faster rates of development are required to produce large or elaborate structures. In these scenarios, the correlation between a life‐history milestone and a structure potentially related to that aspect of life history could serve as a proxy for the effect of development on the shape of the structure.

Horns, antlers, ossicones, and pronghorns (cranial appendages) are characteristic of the clade of even‐toed hoofed mammals known as Pecora (Artiodactyla, Mammalia) sensu Bibi (2014). These cranial appendages are a suitable system for testing the effects of a developmental milestone on shape disparity because their use in intrasexual competition, especially for mating access, is well documented (Emlen, 2008). Cranial appendages follow a common pattern for sexual‐competition structures, in which the complexity of the structure is associated with how it is used in combat or display. Highly complex structures are more likely to be used in nonlethal or ritualized competition, whereas simpler structures can indicate more violent competition (Emlen, 2008). This relationship between shape and fighting style holds true for both bovid horns and cervid antlers (Caro, Graham, Stoner, & Flores, 2003). Further, the size of horns correlates with strength of sexual selection and competition (Bro‐Jørgensen, 2007). The timing of sexual maturity could have an effect on the development and adult shape of pecoran cranial appendages because of these strong connections between their shape and size and the mode and intensity of intrasexual competition.

Several factors may confound the ability to detect a correlation between cranial appendage shape and the age of sexual maturation. First is the potential for a chronological mismatch between sexual maturity and some other measure related to the beginning of sexual competition, like body size. Somatic maturity does not necessarily occur at the same time as sexual maturity, as documented in several vertebrate systems (e.g., Monteiro & Falconer, 1966; Norberg, Weltzien, Karlsen, & Holm, 2001; Wickings & Dixson, 1992). This disconnect in sexual and somatic maturity can be related to sexual bimaturation, in which one sex delays sexual maturity or reproductive effort to achieve some larger body size or competitive skillset (Stamps & Krishnan, 1997). In some taxa, shape change and permanent bone deposition on the cranium continue long after attainment of sexual maturity (Bubenik, 1990). If cranial appendages function as foolproof signals of male quality, continued shape change after reaching sexual maturity is expected: The resource allocation of small, young males toward cranial appendage growth and maintenance should be relatively less than that of larger, older males (Emlen, 2008; Fisher, 1930; Kruuk et al., 2002; Vanpe et al., 2007). The final shape of cranial appendages thus may be significantly different from their shape at sexual maturity, especially in species that mature early and have long life spans. However, the correlation between cranial appendage function, especially fighting style, and shape suggests that continued shape development may improve an individual's ability to compete for mates, but need not dramatically alter the role that appendages of a given shape play in intersexual competition. For example, horns that are used for head butting in animals just reaching sexual maturity may have statistically significant but functionally irrelevant shape differences from fully developed horns.

The importance of sexual maturity as a shape predictor also may be clade specific. The seasonal shedding and regrowth of antlers creates two ontogenetic trajectories: the yearly ontogeny of a new antler developing within a single season and the ontogeny of the antler shape over an individual's lifetime. The annual ontogeny occurs over a fixed period each year, but the rate of shape change increases every year as the antler increases in size and complexity. Variation in this annual ontogeny is likely to be highest between conspecifics. The lifetime ontogeny of antler shape would include only the shape at the end of each growth period, showing overall antler shape changes throughout life rather than just the shape change that occurs as the antler grows anew each season. This lifetime ontogeny would likely vary most between species and could differ in both duration and rate of shape change; the rate of yearly development need not be related to the overall lifetime rate. Although the impermanence of antlers may seem to impede comparisons to the permanent cranial appendages, complex antlers grow to the same shape each year once final adult shape is reached (Whitehead, 1993); thus, comparisons based on life history milestones are valid in the context of the lifetime ontogeny of antler shape.

For female ruminants, the relationship between cranial appendage shape and age at sexual maturity may be even more difficult to ascertain because there is even less consensus as to why females have cranial appendages. Proposed explanations for the presence of female cranial appendages include defense of resources or territories from other females (Espmark, 1971; Holand et al., 2004; Roberts, 1996; Stankowich & Caro, 2009), defense against aggressive males or predators (Bubenik, 1990), and a limited ability to hide due to body size (Stankowich & Caro, 2009). Females do not consistently bear cranial appendages within ruminant families, nor even within species: Some bovid species have appendage‐bearing females only in some populations (Stankowich & Caro, 2009), giraffids and cervids each have only one extant species in which females bear cranial appendages (*Giraffa camelopardalis* and *Rangifer tarandus*, respectively), and the presence of pronghorns in females of the only extant antilocaprid, *Antilocapra americana*, is variable and may be linked to “masculinized” or pseudohermaphrodite females (O'Gara, 1990). However, there is also a link between mutations for hornlessness and hermaphroditism in some breeds of goat (Pailhoux et al., 1994), and in some bovids horn growth is greatest when sex hormones are at their lowest, with the growth rates significantly reduced once an animal reaches sexual maturity (Bubenik, 1990). This connection between cranial appendage presence and sexual development, along with the sexual dimorphism of cranial appendage shapes in some taxa (Bubenik, 1990), requires the testing of the effect of age at sexual maturity on cranial appendage shape not just for males but for females as well.

Two aspects of cranial appendage evolution could benefit from a better understanding of their development: the reason for their diversity and their homology. Although there is a strong functional link between cranial appendages and intrasexual competition, whether their use as weaponry is the reason for their evolution and diversity is debated. Recent studies of cranial appendage evolution propose three main explanations for structural and shape diversity: species recognition, habitat controls, and sexual selection. Some researchers argue that cranial appendage diversity is related to the need for animals to expend reproductive effort only on members of the same species (Vrba, 1984), although there is minimal evidence that female mate‐choice influences cranial appendage shape (Caro et al., 2003). Likewise, if males use cranial appendages as cues for mate choice, the lack of cranial appendages in many ruminant females would prevent the application of this theory to all ruminants. Multiple studies have focused on the relationship between size and complexity of cranial appendages and the degree of habitat openness (Emlen, 2008; Janis, 1990; Jarman, 1974; Packer, 1983), finding support for correlations between body size, habitat variation, and the size of cranial appendages. The popularly accepted function of cranial appendages is as weapons for intrasexual competition between males over mate access (Barette & Vandal, 1990; Bartos & Bahbouh, 2006; Clutton‐Brock, 1982; Emlen, 2008). Although this explanation would appear to have the most support, it does not consider females that have cranial appendages (Emlen, 2008; Kiltie, 1985). Shape diversity driven solely by female choice rather than selection in sexual competition is expected to produce completely random shapes, but there is little support for complete randomness of cranial appendage shapes, and the correlation between shape and fighting strategy is well supported (Caro et al., 2003).

Studies of the cranial appendage homology may also be improved with a developmental focus. The families of Pecora possessing cranial appendages (the antilocaprids, giraffids, bovids, and cervids) appear in the fossil record in the early Miocene (between 23 to 16 Ma), and the earliest representatives of these families have their respective cranial appendage types, that is, antilocaprids have pronghorns, giraffids have ossicones, bovids have horns, and cervids have antlers (Bibi, 2013; DeMiguel, Azanza, & Morales, 2014). The rapid radiation of Pecora over this period resulted in rampant homoplasy of morphological characters (Bibi, 2014; Hassanin & Douzery, 2003; Kraus & Miyamoto, 1991); thus, cranial appendages are often used to diagnose familial relationships because they offer one of the strongest synapomorphies for each family (Davis, Brakora, & Lee, 2011). The permanent bone portions of cranial appendages are often the only part of the structure preserved, however, and each type of cranial appendage is composed of some permanent bone portion, complicating familial assignments for some fossils due to the similarity of these bony cores (Bibi et al., 2009). That some female pecorans lack cranial appendages exacerbates this problem; any fossil pecoran without cranial appendages may be a female of a species with appendage‐bearing males, rather than a representative of a taxon without cranial appendages in either sex (Bibi et al., 2009). Until cranial appendage‐lacking fossil taxa are confidently placed at the base of the Pecoran families, research on the homology of cranial appendages may be better served by studies of their development (Davis et al., 2011).

To date, no studies have attempted to provide developmental explanations for ruminant cranial appendage shape disparity. The functional link between cranial appendages and sexual competition strongly implies that sexual maturity could be a milestone of interest in early research on the connection between cranial appendage ontogeny and the evolution of their disparity. In this study, therefore, I tested the hypothesis that the average age at which animals reach sexual maturity, defined as showing some sign of sexual reproductive capability, is correlated with the size or shape of cranial appendages. I predict that taxa with delayed onset of sexual maturity have extended the development of their cranial appendages beyond that of ancestral taxa, resulting in more elaborate cranial appendages (e.g., more tines, crenulations, spirals).

## 2 Methods

**Figure 1:**
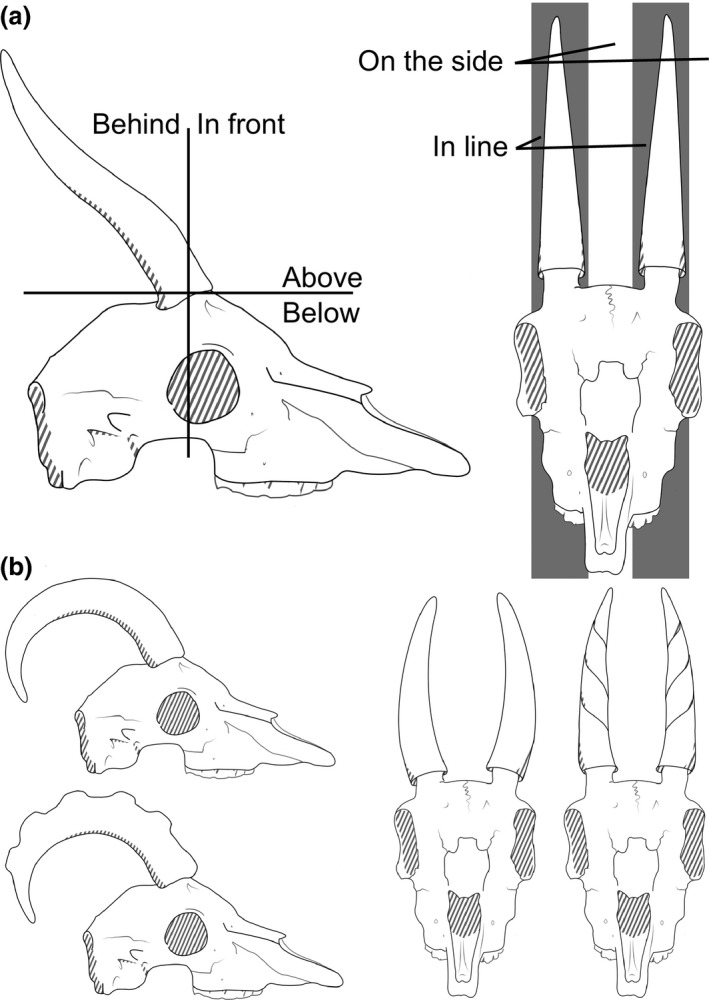
Cartoons of the shape variables used in this study. Part a shows the variables that describe the position of the tip relative to the base of the cranial appendage. Part b shows smooth versus crenulated appendages in the lateral views on the left and straight versus twisted cranial appendages in anterior views on the right

Cranial appendage shapes, previously encoded as presence/absence categorical variables for a study of ecological and behavioral correlations with shape in bovids and cervids (Caro et al., 2003), comprise the shape data in this study. These data capture qualitative shape information in three groups of variables: the position of the tip relative to the base, the surface characteristics of the appendages, and the number of tines on each appendage (in three bins: one tine, two to five tines, greater than five tines). The first two groups of variables are coded in oppositional pairs, such as tips that end anterior to the cranial appendage base or posterior to the appendage base (Figure 1). For males, these paired variables are mutually exclusive, so that a taxon coded as “present” for one variable will be coded “absent” in its pair. For females without cranial appendages and both sexes of *Moschus moschiferus*, all variables are coded as “absent.” The average lengths of adult cranial appendages were collected from literature for males and females. Body mass data were also compiled for males and females to account for its effect on cranial appendage size and shape in analyses. For bovids, horn lengths were reported without respect to how they were measured (Bro‐Jørgensen, 2007), a potential source for inconsistencies in that data. All antler lengths are measured along the main beam (Plard, Bonenfant, & Gaillard, 2011). The studies from which those data were compiled focused only on Bovidae and Cervidae, but because understanding the evolutionary history of these characters requires at least a three‐taxon comparison, literature data were collected for as many variables as possible for *A. americana*,* G. camelopardalis*, and *M. moschiferus*, to represent Antilocapridae, Giraffidae, and Moschidae (see Supporting information S1–S5 for data collected and complete sources). The author measured the average ossicone length from the apex of the right ossicone to the anterior‐most point of the suture between the ossicone and the frontal bone on four female *G. camelopardalis* crania (AMNH 35628, AMNH 83458, AMNH 53549, AMNH 53546), because female ossicone length data were not available in published literature.

Sexual maturity is reported in the literature in several ways, for example, age at first pregnancy, first birth, first ovulation, first semen production, first siring, and first mating. For some taxa, the only data available are reported as “sexual maturity” without reference to a specific physiological change, these data were included as two variables, “male sexual maturity” and “female sexual maturity.” All reported ages were transformed into months after birth. When ranges of ages were provided, the median value was used. When sample sizes were provided, values found in at least 50% of the animals in the study were used. Widely domesticated animals were not used in the study, as these animals are often bred to have certain cranial appendage shapes and sizes, and to reproduce earlier in their life cycles, as in Bro‐Jørgensen (2007).

The average age at sexual maturity was calculated from the available variables for each taxon and then divided by the average life span of that species reported in Myhrvold et al. (2015). Dividing by life span represents the age at sexual maturity as a relative measure of how much of the animal's life has elapsed by the time it reaches sexual maturity. This proportional age variable and the natural log‐transformed body mass variable for each taxon were standardized to have means of zero and standard deviations of one. This standardizing step can improve the fit of statistical models and makes the value of the intercept easier to interpret. Interpretation of the intercept is often difficult because it represents the value of the dependent variable when all independent variables equal zero, a state that is often meaningless for biological data. By centering the means of the independent variables on zero, the intercept can be interpreted as the value of the dependent variable when each independent variable holds the average value (Gelman & Hill, 2007).

Phylogenetically informed statistical tests were performed using a maximum‐likelihood topology from an analysis of complete mitochondrial genomes for 210 taxa that implemented uniform priors on divergence dates and hard fossil calibrations to model branch lengths (Hassanin et al., 2012). All taxa from that phylogeny that are not represented in the final matrix of categorical and continuous data were pruned from the tree, resulting in a tree with 69 taxa: 46 bovids, 20 cervids, and one taxon each from the other extant pecoran families (Moschidae, Giraffidae, and Antilocapridae). The full phylogeny included subspecies‐level data as well as multiple individuals for some taxa; however, published subspecies‐level data for shape, sexual maturity, and body mass are not readily available; thus, most of these taxa were pruned in instances where doing so would not require an ad hoc choice for their placement in the phylogeny. Taxon names from Hassanin et al. (2012) remain unchanged so that the replicate or subspecies from the original phylogeny can be identified. When duplicate taxa could not be removed because their position in the phylogeny was ambiguous, the data available for those species were repeated for all replicates.

Each variable describing cranial appendage shape or size was tested for correlation with the average age of sexual maturity. Shape variables for males and females were tested separately for correlation with average age at sexual maturity, accounting for the average body mass reported for that sex and the interaction between sexual maturity and mass as modeled by the linear equation below (Equation (1)): *S* equals size or a binary shape variable, *M* equals average sexual maturity, BM is body mass, and *b* is the intercept.

(1) S=M+BM+M:BM+b

Phylogenetic generalized least‐squares (PGLS) analyses were used to test correlations between cranial appendage length and sexual maturity. A correlation structure was calculated based on the pruned phylogeny and allowing the function corPagel (R package ape) to estimate the best value of Pagel's lambda from an initial starting lambda of 0.5 (Paradis, Claude, & Strimmer, 2004; Pinheiro, Bates, DebRoy, Sarkar, & R Core Team, 2015). Ninety‐five percent confidence intervals were computed for all coefficients obtained by PGLS (intervals, R package nlme; Pinheiro et al., 2015). PGLS is not suitable for analyses of binary dependent variables, because it assumes the residuals will have a normal distribution and that the data can take any value rather than just the two binary values (Ives & Garland, 2014). A more appropriate formulation for analysis of such data is the generalized linear mixed‐effects model (GLMM), which models an underlying continuous trait that provides the probability of the binary variable being either state (Ives & Garland, 2014).

Binary shape variables were tested using a GLMM that converted between this underlying continuous trait and the binary probabilities with an inverse logit link function using the function binaryPGLMM (R package ape; Paradis et al., 2004). BinaryPGLMM estimates model parameters by alternately estimating the coefficients under penalized quasi‐likelihood and the mixed‐effect variances using restricted maximum likelihood until convergence between the two is reached (Ives & Helmus, 2011). GLMM 95% confidence intervals were generated using a 1000‐replicate parametric bootstrap. Analyses were repeated for males and females using the full dataset, a subsample with cervids removed, and a subsample with bovids removed to see whether the differing growth modes of horns and antlers would affect detection of correlations. The subsample with bovids removed did not have sufficient sample numbers to achieve convergence for the binary shape GLMM tests for males or females or the PGLS for female cranial appendage length. Similarly, because horns are unbranched and only one female cervid has antlers (*R. tarandus*), the shape variables describing cranial appendages with greater than one tine could not be analyzed for females or for the male and female subsamples with the cervids removed.

## 3 Results

### 3.1 Males

**Figure 2:**
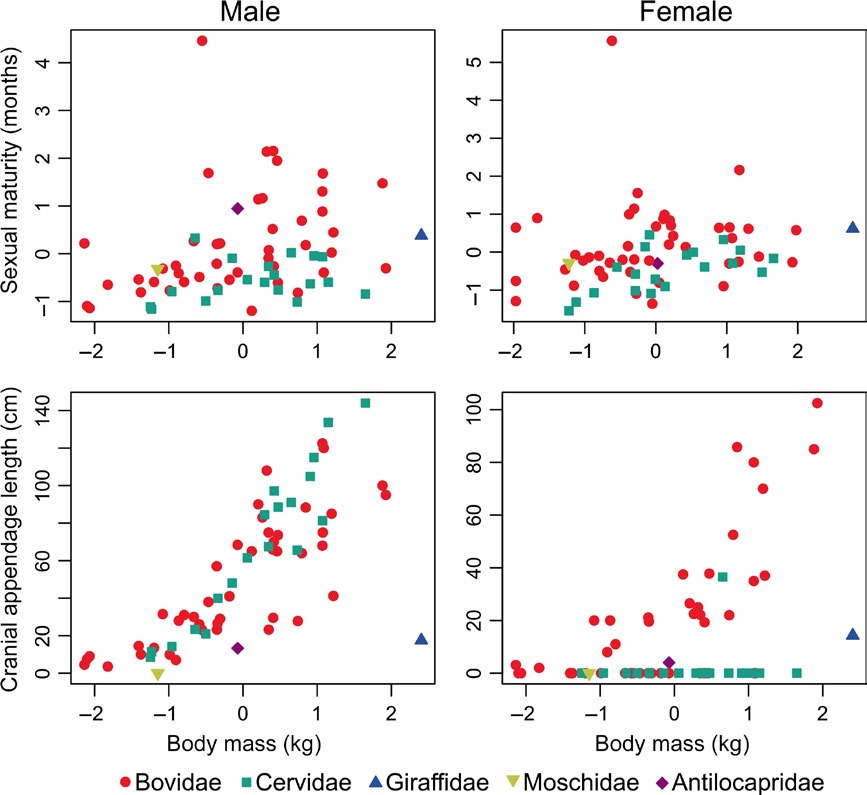
Scatterplots showing uncorrelated age at sexual maturity and body mass and correlated cranial appendage length and body mass for males and females

The effect of phylogeny on size and shape of male cranial appendages was significant in all models analyzed for the full taxon sample (Table 1), even when there was no correlation between shape and the other independent variables. Body mass had a significant effect on cranial appendage length (Figure 2) and on the lateral or coplanar position of cranial appendage tips relative to their bases. When age at sexual maturity is at its average value across all taxa (28 months for males), an increase of one standard deviation in body mass would result in a 30‐centimeter increase in length of male cranial appendages. Raising body mass by one standard deviation also increases the probability of cranial appendages with tips ending to the side of their bases and decreases the probability of cranial appendages with tips and bases in the same plane by approximately 0.3. No shape variable correlated with age at sexual maturity alone; however, the interaction between age at sexual maturity and body mass was significantly correlated with whether appendages have one tine or two to five tines. The significant interaction terms in these models mean that the effect of body mass on the probability of having cranial appendages with only one tine or with two to five tines changes at different ages of sexual maturity.

**Table 1:** Results for shape variables tested for all males, with 95% confidence intervals for each coefficient and significant coefficients in bold

	Phylogenetic effect	Intercept	Sexual maturity	Body mass	Maturity: mass
Length	**0.830 (0.564, 0.830)**	**31.482 (3.914, 59.051)**	−1.211 (−7.632, 5.210)	**30.259 (22.802, 30.259)**	−1.929 (−9.300, 5.442)
Tip position					
Above	**4.932 (5.365, 9.864)**	**3.055 (2.477, 5.380)**	−0.239 (−1.501, 0.488)	0.228 (−0.765, 1.122)	0.139 (−1.200, 0.987)
Below	**5.540 (6.385, 11.008)**	**−3.976 (−6.851, −4.156)**	0.301 (−0.455, 1.664)	0.349 (−0.600, 1.466)	−0.264 (−1.225, 1.030)
In front	**2.722 (1.468, 5.444)**	**−2.432 (−4.191, −1.439)**	−0.068 (−0.722, 1.326)	−0.016 (−0.924, 0.948)	−0.114 (−0.985, 1.136)
Behind	**2.547 (1.283, 5.095)**	**2.780 (1.815, 4.560)**	0.367 (−1.315, 1.048)	0.204 (−0.867, 1.100)	0.077 (−1.471, 1.001)
On side	**4.952 (5.573, 9.903)**	−1.260 (−3.130, 0.342)	0.406 (−0.258, 1.304)	**1.415 (0.551, 2.416)**	0.054 (−1.021, 1.013)
In line	**4.717 (5.324, 9.434)**	1.404 (−0.005, 3.241)	−0.297 (−1.100, 0.354)	**−1.225 (−2.131, −0.399)**	−0.109 (−0.950, 0.784)
Surface					
Smooth	**5.948 (8.037, 11.896)**	0.315 (−1.206, 2.034)	0.290 (−0.455, 1.024)	0.644 (−0.017, 1.475)	0.190 (−0.67, 1.007)
Crenulated	**5.940 (7.945, 11.88)**	−0.709 (−2.356, 0.942)	−0.288 (−1.003, 0.552)	−0.461 (−1.234, 0.203)	−0.186 (−1, 0.665)
Straight	**5.638 (7.251, 11.275)**	1.096 (−0.265, 2.922)	−0.298 (−1.109, 0.336)	−0.303 (−1.106, 0.381)	0.159 (−0.715, 0.912)
Twisted	**5.783 (7.364, 11.565)**	−1.560 (−3.56, −0.413)	0.315 (−0.409, 1.12)	0.537 (−0.256, 1.372)	−0.198 (−1.021, 0.666)
Tines					
1	**7.605 (10.787, 15.211)**	0.885 (−0.543, 2.920)	0.777 (−0.064, 1.780)	−0.171 (−1.195, 0.624)	**2.301 (1.226, 3.879)**
2–5	**6.471 (8.415, 12.941)**	**−1.930 (−4.098, −0.814)**	−0.446 (−1.328, 0.850)	−0.509 (−1.506, 0.422)	**−1.532 (−2.84, −0.291)**
Greater than 5	**3.391 (2.223, 6.781)**	**−3.849 (−6.202, −3.060)**	−0.063 (−0.807, 1.643)	1.137 (−0.088, 2.167)	−0.671 (−1.829, 0.665)

When bovids or cervids were removed from the sample, the effect of body mass on cranial appendage length remained significant, although the magnitude of the effect was different. With bovids removed, a change of one unit standard deviation in body mass would result in a 35‐centimeter change in cranial appendage length assuming age at sexual maturity was average (Table 2); however, this model also had a significant interaction between sexual maturity and body mass, meaning that for cervids the effect of body mass on cranial appendage length differs depending on the age at sexual maturity of the taxon in question. For the sample with cervids removed, changing body mass by one standard deviation would result in a 23‐centimeter change in cranial appendage length when sexual maturity is at the average value. There was no significant interaction between sexual maturity and body mass for bovids; thus, the effect of body mass on horn length does not differ depending on age at sexual maturity. There were also no significant correlations between sexual maturity or body mass and the binary shape variables when cervids were removed from the analyses.

**Table 2:** Results for shape variables tested for bovid males and cranial appendage length for bovid males and cervid males, with 95% confidence intervals for each coefficient and significant coefficients in bold

	Phylogenetic effect	Intercept	Sexual maturity	Body Mass	Maturity : mass
Cervid length	**0.723 (0.161, 1.284)**	**43.307 (10.957, 75.657)**	−10.098 (−22.329, 2.133)	**35.089 (24.131, 46.047)**	**−12.700 (−23.215, −2.184)**
Bovid length	**0.845 (0.444, 1.246)**	23.820 (−4.808, 52.449)	1.135 (−5.893, 8.162)	**23.299 (13.989, 32.808)**	2.649 (−6.270, 11.569)
Bovid shape					
Tip position					
Above	**4.789 (5.170, 9.577)**	**2.515 (2.034, 4.975)**	−0.165 (−1.299, 0.643)	0.206 (−0.995, 1.143)	0.261 (−1.385, 1.273)
Below	**5.491 (6.241, 10.982)**	**−3.539 (−6.519, −3.740)**	0.261 (−0.596, 1.507)	0.356 (−0.759, 1.609)	−0.423 (−1.592, 1.301)
In front	**2.239 (0.792, 4.478)**	**−1.991 (−3.668, −0.947)**	−0.249 (−0.985, 1.050)	0.027 (−0.961, 1.193)	−0.311 (−1.359, 1.287)
Behind	**1.890 (0.038, 3.781)**	**2.372 (1.238, 3.942)**	0.634 (−1.295, 1.432)	0.130 (−1.169, 1.078)	0.209 (−1.520, 1.306)
On side	**3.736 (3.092, 7.471)**	**−1.759 (−3.738, −0.240)**	0.532 (−0.249, 1.550)	1.016 (−0.237, 1.949)	0.565 (−0.631, 1.743)
In line	**3.503 (2.827, 7.005)**	**1.894 (0.501, 3.766)**	−0.407 (−1.583, 0.262)	−0.814 (−1.681, 0.325)	−0.663 (−1.782, 0.548)
Surface					
Smooth	**5.013 (5.766, 10.026)**	−0.827 (−2.663, 0.984)	0.488 (−0.312, 1.354)	0.826 (−0.220, 1.768)	0.226 (−0.929, 1.214)
Crenulated	**5.073 (6.207, 10.146)**	0.266 (−1.372, 2.030)	−0.451 (−1.347, 0.397)	−0.542 (−1.504, 0.326)	−0.284 (−1.369, 0.715)
Straight	**5.123 (6.288, 10.246)**	0.368 (−1.177, 2.079)	−0.205 (−1.021, 0.688)	−0.371 (−1.295, 0.518)	0.357 (−0.647, 1.334)
Twisted	**5.251 (6.249, 10.501)**	−0.886 (−2.829, 0.699)	0.234 (−0.696, 1.210)	0.618 (−0.324, 1.559)	−0.415 (−1.503, 0.739)
Tines					
1	**6.404 (8.411, 12.808)**	**2.399 (1.852, 4.948)**	−0.148 (−1.353, 0.696)	0.945 (−0.448, 2.087)	0.417 (−1.554, 1.447)
2–5	–	–	–	–	–
Greater than 5	–	–	–	–	–

**Figure 3:**
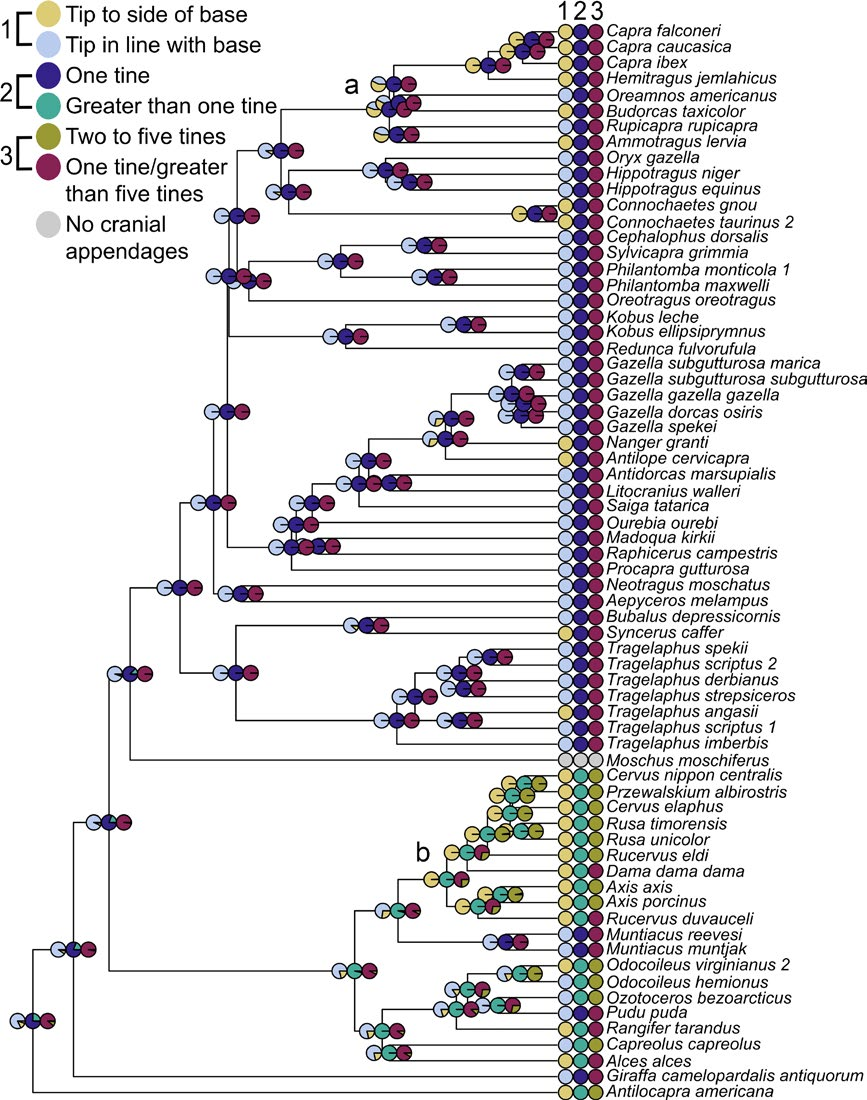
Ancestral state reconstructions of male cranial appendage shape variables that had significant correlations with body mass or the interaction between body mass and age at sexual maturity

The ancestral state reconstructions of the three shape variables that correlated with body mass are simple for bovids but more complex for cervids. Horns in most bovid taxa have their tips in line with their bases. Horns with tips to the side of the bases arose unambiguously at the base of the tribe Caprini (here represented by the genera *Capra* and *Hemitragus*) and may have been present in the ancestor of Caprinae (Figure 3a). Horns with tips lateral to their bases appear to have evolved separately in several other clades across Bovidae as well. All horns are unbranched; thus, the ancestor of all bovids reconstructed to have unbranched cranial appendages (Figure 3.2, 3.3). Antlers with tips lateral to their bases reconstructed with one major radiation in the Cervini (Figure 3b) and have appeared in various other clades. The ancestor of all cervids reconstructed with antler tips that are in line with their bases and greater than one tine (Figure 3.2), but also as having either one tine or greater than five tines (Figure 3.3). Taken together, these last two variables suggest an ancestor with cranial appendages that had greater than five tines, although early deer fossils have simple antlers with only two to three tines (Bubenik, 1990).

### 3.2 Females

As with males, phylogeny was a significant predictor of cranial appendage length and all shape variables in females (Table 3). Body mass was a significant predictor of cranial appendage length, corresponding to an increase in length of 13 centimeters for every standard deviation increase in body mass when age at sexual maturity is at its average (22 months for females). Body mass was a significant predictor of several binary cranial appendage shape variables. At the average age of female sexual maturation, a single standard deviation increase in body mass increases the probability of having appendages with tips ending above and behind their bases by 0.2 and the probability of tips lateral to their bases by 0.1. A standard deviation increase in body mass also corresponds to an increase in the probability of smooth cranial appendages by 0.2 and of twisted cranial appendages by 0.1. For crenulated cranial appendages, the interaction between age at sexual maturity and body mass was significant. Lastly, body mass correlated with the presence of cranial appendages with only one tine, the probability of which increases by 0.2 for every standard deviation increase in body mass.

**Table 3:** Results for shape variables tested for all females, with 95% confidence intervals for each coefficient and significant coefficients in bold

	Phylogenetic effect	Intercept	Sexual maturity	Body Mass	Maturity : mass
Length	**0.751 (0.413, 1.089)**	7.261 (−12.744, 27.265)	−2.147 (−7.551, 3.257)	**13.083 (7.626, 18.541)**	1.119 (−5.040, 7.277)
Tip position					
Above	**3.057 (2.743, 6.115)**	−0.574 (−1.895, 0.834)	−0.220 (−0.961, 0.598)	**0.901 (0.211, 1.603)**	−0.537 (−1.454, 0.332)
Below	**6.096 (7.466, 12.193)**	**−3.896 (−6.733, −4.105)**	−0.279 (−1.018, 1.179)	0.140 (−0.759, 1.062)	−0.187 (−1.083, 1.003)
In front	**2.808 (1.516, 5.616)**	**−2.999 (−4.911, −2.192)**	−0.671 (−1.529, 0.705)	0.445 (−0.446, 1.325)	0.250 (−0.580, 1.562)
Behind	**4.358 (5.004, 8.716)**	−0.470 (−2.082, 1.064)	−0.308 (−1.075, 0.464)	**0.905 (0.327, 1.698)**	−0.730 (−1.599, 0.062)
On side	**4.598 (5.138, 9.195)**	**−1.961 (−3.908, −0.811)**	−0.150 (−0.791, 0.875)	**0.877 (0.171, 1.735)**	−0.059 (−1.010, 0.883)
In line	**3.367 (3.305, 6.734)**	−0.905 (−2.522, 0.412)	−0.358 (−1.106, 0.454)	0.451 (−0.137, 1.072)	−0.737 (−1.619, 0.115)
Surface					
Smooth	**2.954 (2.030, 5.908)**	**−1.969 (−3.615, −0.475)**	0.530 (−0.345, 1.555)	**1.019 (0.136, 1.787)**	0.001 (−1.019, 1.087)
Crenulated	**4.313 (4.374, 8.625)**	**−1.940 (−3.796, −0.716)**	−0.946 (−1.743, 0.192)	0.452 (−0.282, 1.297)	**−1.065 (−2.224, −0.114)**
Straight	**3.411 (3.221, 6.821)**	**−1.384 (−2.867, −0.084)**	−0.258 (−0.950, 0.690)	0.559 (−0.134, 1.231)	−0.325 (−1.165, 0.612)
Twisted	**6.393 (8.453, 12.786)**	**−2.316 (−4.578, −1.304)**	−0.112 (−0.924, 0.960)	**0.929 (0.186, 1.796)**	−0.680 (−1.785, 0.315)
Tines					
1	**5.153 (6.109, 10.305)**	−1.247 (−3.100, 0.297)	−0.375 (−1.087, 0.563)	**1.042 (0.331, 1.905)**	−0.623 (−1.577, 0.264)
2–5	–	–	–	–	–
Greater than 5	–	–	–	–	–

Removing cervids from the analyses of shape variables produced correlations similar to those found in the full sample, although the strength of the effect varies (Table 4). Increasing body mass by one standard deviation corresponds to an 18‐centimeter increase in cranial appendage length when cervids are not present in the sample. The correlation between body mass and tips that end above their base was no longer significant, but the effect of a single standard deviation increase in body mass on the probability of tips behind the cranial appendage base and tips lateral to the base remained 0.2 and 0.1, respectively. The correlation between body mass and smooth cranial appendages remained 0.2, but the effect of body mass on the probability of twisted cranial appendages increased from 0.1 to 0.2 with cervids removed. The interaction between age at sexual maturity and body mass that was significant for crenulated cranial appendages was no longer significant in the reduced sample. The effect of body mass on the presence of single‐tined cranial appendages increased from a 0.2 to a 0.3 increase in probability per standard deviation of change in body mass.

**Table 4:** Results for shape variables tested for bovid females, with 95% confidence intervals for each coefficient and significant coefficients in bold

	Phylogenetic effect	Intercept	Sexual maturity	Body Mass	Maturity : mass
Length	**0.592 (0.121, 1.063)**	9.461 (−10.117, 29.038)	−3.989 (−10.434, 2.457)	**18.033 (11.027, 25.038)**	−2.759 (−11.103, 5.585)
Tip position					
Above	**2.044 (0.606, 4.088)**	−0.042 (−1.617, 1.316)	−0.448 (−1.257, 0.610)	0.977 (−0.093, 1.722)	−0.953 (−2.012, 0.625)
Below	**5.985 (7.496, 11.970)**	**−3.532 (−6.553, −3.682)**	−0.400 (−1.177, 1.143)	0.143 (−0.779, 1.215)	−0.280 (−1.463, 1.215)
In front	**2.068 (0.269, 4.135)**	**−2.826 (−4.623, −1.940)**	−1.049 (−2.086, 0.491)	0.557 (−0.360, 1.676)	0.231 (−0.898, 1.885)
Behind	**4.060 (4.226, 8.121)**	0.106 (−1.472, 1.875)	−0.563 (−1.485, 0.461)	**0.974 (0.050, 1.884)**	−1.261 (−2.473, 0.231)
On side	**5.241 (6.274, 10.481)**	**−1.841 (−3.960, −0.626)**	−0.376 (−1.192, 0.806)	**0.987 (0.120, 1.983)**	−0.143 (−1.318, 1.061)
In line	**1.935 (0.667, 3.869)**	−0.392 (−1.655, 0.961)	−0.489 (−1.283, 0.527)	0.382 (−0.383, 1.138)	−1.023 (−2.058, 0.319)
Surface					
Smooth	**3.307 (2.294, 6.615)**	**−1.729 (−3.59, −0.080)**	0.462 (−0.722, 1.531)	**1.206 (0.046, 2.110)**	−0.172 (−1.520, 1.406)
Crenulated	**3.248 (2.553, 6.495)**	−1.286 (−2.951, 0.218)	−1.072 (−1.894, 0.437)	0.351 (−0.623, 1.348)	−1.416 (−2.682, 0.217)
Straight	**3.101 (2.845, 6.201)**	−1.003 (−2.566, 0.399)	−0.443 (−1.171, 0.694)	0.547 (−0.249, 1.385)	−0.525 (−1.643, 0.762)
Twisted	**6.225 (8.102, 12.451)**	**−1.851 (−4.099, −0.867)**	−0.188 (−1.143, 1.001)	**0.920 (0.142, 2.013)**	−0.968 (−2.347, 0.268)
Tines					
1	**4.139 (4.384, 8.278)**	−0.469 (−2.264, 1.149)	−0.553 (−1.496, 0.658)	**1.137 (0.178, 2.028)**	−1.049 (−2.395, 0.408)
2–5	–	–	–	–	–
Greater than 5	–	–	–	–	–

**Figure 4:**
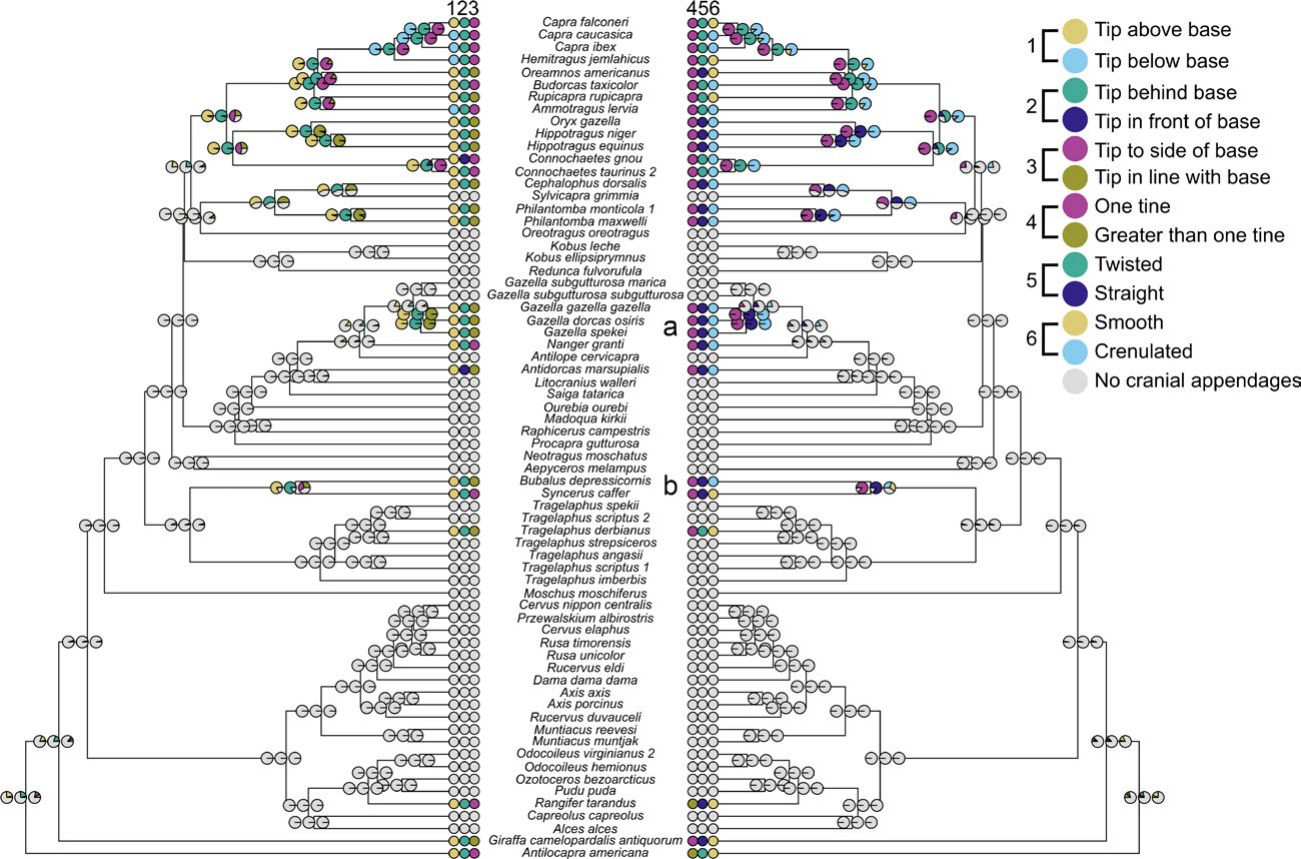
Ancestral state reconstructions of female cranial appendage shape variables that had significant correlations with body mass or the interaction between body mass and age at sexual maturity

The main radiation of cranial appendage‐bearing females is the clade composed of the tribes Caprini, Hippotragini, and Alcelaphini, and the ancestral state reconstructions for their common ancestor conform to the states for these characters found in most extant females. Females of this hypothetical ancestor likely had cranial appendages with tips ending above, behind, and to the side of their bases, with twisting and crenulated surfaces. The majority of female cranial appendages have tips that are in line with their bases (Figure 4.3) and have straight, as opposed to twisted, surfaces (Figure 4.5). Given that there are only three females that bear cranial appendages outside of Bovidae, and only two of those females (*R. tarandus* and *A. americana*) have cranial appendages with more than on tine, the reconstruction for one‐tined appendages (Figure 4.4) can represent not only this particular shape variable but also the presence or absence of cranial appendage‐bearing females throughout Pecora. Females with cranial appendages appear at the base of the Caprini + Hippotragini + Alcelaphini clade, in several clades within the Antilopini (Figure 4a), and within the Bovini (Figure 4b).

## 4 Discussion

Most of the tests showed no significant correlation between average age of sexual maturity and shape variables. Although the prediction was that structures with a potential evolutionary link and observed functional link to sexual selection would correlate with such an important life history and sexual reproduction milestone as the age at which reproduction is first possible, when correlations were found they were between shape or size and body mass instead. Differences in cranial appendage growth patterns across clades, as well as the mismatch between when shape stops developing and when sexual maturity is reached, could account for the decoupling of sexual maturity and shape variation.

Cranial appendages are some of the most elaborate and diverse skeletal structures generated by mammals, which is reflected in these data when the scorings for all variables are compared between species. Analyzing each shape variable independently reduced the variation captured to a simple presence/absence binary rather than encompassing the full suite of variation between appendages. Studying shape with binary characters obviated the time demands and analytical complexity required by methods such as geometric morphometrics at the expense of capturing the complete range shape variation in cranial appendages. Furthermore, atomizing each variable into even more discrete states would result in meaningless statistical tests, as there would be few exemplars of any particular state exhibited across the potential taxon sampling size. Thus, the explanatory value of sexual maturity may be diminished in this study by the low variation in binary variables when they are analyzed in isolation.

Caro et al. (2003) also found associations between cranial appendage shape and body mass. They found that male bovids with larger body sizes were more likely to have downward pointing tips and that large females were more likely to have downward pointing, smooth, crenulated, or twisted cranial appendages. They also found that upward pointing smooth horns are more common in small‐bodied males. This study also found that smooth and twisted cranial appendages are more common in larger females, but did not find a clear relationship between crenulations and body size. Likewise, this study found that upward pointing cranial appendages are more common in large females rather than small females. The results presented here are not directly comparable given differences in phylogeny, use of a different comparative method, and the use of discretized body mass in Caro et al. (2003).

The importance of body mass as a predictor of cranial appendage shape rather than age at sexual maturity does not obviate developmental explanations for cranial appendage shape disparity, nor does it suggest that it is impossible to detect developmental explanations for shape disparity using a milestone such as age at sexual maturity. The relationship between cranial appendage shape and body mass suggests that a more appropriate developmental milestone for cranial appendages may be the age at which animals reach average adult body mass. Without further testing, however, the most rigorous way to test the evolution and development of disparity remains through the use of ontogenetic series.

## Conflict of Interest

None declared.
