# Supplementary material for: Sexual maturity and shape development in cranial appendages of extant ruminants
Source: Ecol Evol. 2016 Oct 9;6(21):7820–30. doi: 10.1002/ece3.2512 (PMC6093164; doi:10.1002/ece3.2512)
Supplement: Supplementary file 1 [file ECE3-6-7820-s001.docx]

**Appendices**

**Sexual maturity data**

All sexual maturity variables presented here are the ages of first occurrence of specific structures or events (e.g., first corpus luteum, first conception), converted to months after birth and rounded to the nearest whole month. Citations for each cell are in square brackets following the value.

**Appendix S1: Male sexual maturity**

|  | Age (months after birth) at first: | | | | | |
| --- | --- | --- | --- | --- | --- | --- |
|  | Large testes | Spermatogenesis | Sperm | Mating | Sire | "Sexual Maturity" |
| *Alces alces* | NA | NA | NA | 14^[12]^ | NA | 21^[12, 14]^ |
| *Axis axis* | NA | NA | NA | NA | 15^[10]^ | 17^[14]^ |
| *Axis porcinus* | NA | NA | NA | NA | 10^[10]^ | 14^[14]^ |
| *Blastocerus dichotomus* | NA | NA | NA | NA | NA | NA |
| *Capreolus capreolus* | NA | NA | NA | 36^[12]^ | NA | 14^[12, 14]^ |
| *Cervus elaphus* | 48^[12]^ | NA | 12^[12]^ | NA | 12^[12]^ | 22^[12, 14]^ |
| *Cervus nippon centralis* | NA | NA | NA | 42^[12]^ | NA | 17^[14]^ |
| *Cervus nippon taiouanus* | NA | NA | NA | 42^[12]^ | NA | 17^[14]^ |
| *Cervus nippon yakushimae* | NA | NA | NA | 42^[12]^ | NA | 17^[14]^ |
| *Dama dama dama* | 13^[12]^ | 10^[12]^ | 15^[12]^ | NA | NA | 18^[12, 14]^ |
| *Dama dama mesopotamica* | 13^[12]^ | 10^[12]^ | 15^[12]^ | NA | NA | 18^[12, 14]^ |
| *Elaphodus cephalophus* | NA | NA | NA | NA | NA | 21^[14]^ |
| *Elaphurus davidianus* | NA | NA | NA | NA | NA | NA |
| *Hydropotes inermis* | NA | NA | NA | NA | 7^[10]^ | 10^[14]^ |
| *Mazama gouazoubira* | NA | NA | NA | NA | NA | 22^[14]^ |
| *Mazama rufina* | NA | NA | NA | NA | NA | 12^[9]^ |
| *Muntiacus crinifrons* | NA | NA | NA | NA | NA | NA |
| *Muntiacus muntjak* | NA | NA | NA | NA | NA | 12^[12, 14]^ |
| *Muntiacus reevesi* | NA | NA | NA | NA | 6^[10]^ | 10^[14]^ |
| *Muntiacus vuquangensis* | NA | NA | NA | NA | NA | NA |
| *Odocoileus hemionus* | NA | NA | NA | 15^[12]^ | 12^[12]^ | 16^[12, 14]^ |
| *Odocoileus virginianus 1* | NA | NA | NA | 11^[12]^ | 14^[12]^ | 12^[14]^ |
| *Odocoileus virginianus 2* | NA | NA | NA | 11^[12]^ | 14^[12]^ | 12^[14]^ |
| *Odocoileus virginianus 3* | NA | NA | NA | 11^[12]^ | 14^[12]^ | 12^[14]^ |
| *Ozotoceros bezoarcticus* | NA | NA | NA | NA | NA | 12^[9]^ |
| *Przewalskium albirostris* | NA | NA | NA | NA | NA | 24^[16]^ |
| *Pudu mephistophiles* | NA | NA | NA | NA | NA | 12^[9]^ |
| *Pudu puda* | NA | NA | NA | NA | 9^[12]^ | 7^[9]^ |
| *Rangifer tarandus* | 84^[12]^ | NA | 18^[12]^ | 27^[12]^ | 29^[10]^ | 23^[12, 14]^ |
| *Rucervus duvauceli* | NA | NA | NA | NA | 37^[10]^ | 28^[14]^ |
| *Rucervus eldi* | NA | NA | NA | NA | NA | 18^[14]^ |
| *Rusa timorensis* | NA | NA | NA | NA | NA | 22^[14]^ |
| *Rusa unicolor* | NA | NA | NA | NA | NA | 21^[14]^ |
| *Moschus moschiferus* | NA | NA | NA | NA | NA | 13^[12, 14]^ |
| *Addax nasomaculatus* | NA | NA | NA | NA | NA | NA |
| *Aepyceros melampus* | NA | 16^[12]^ | NA | NA | 13^[12]^ | 17^[14]^ |
| *Alcelaphus buselaphus buselaphus* | NA | NA | NA | NA | NA | 28^[14]^ |
| *Alcelaphus buselaphus lichtensteinii* | NA | NA | NA | NA | NA | 28^[14]^ |
| *Ammotragus lervia* | NA | NA | NA | NA | NA | 17^[14]^ |
| *Antidorcas marsupialis* | NA | NA | 12^[12]^ | 18^[12]^ | NA | 11^[14]^ |
| *Antilope cervicapra* | NA | NA | NA | NA | NA | 23^[14]^ |
| *Arabitragus jayakari* | NA | NA | NA | NA | NA | NA |
| *Bison bison* | NA | NA | NA | 33^[12]^ | NA | 31^[12, 14]^ |
| *Bison bonasus* | NA | NA | NA | NA | NA | 29^[12, 14]^ |
| *Bos gaurus* | NA | NA | NA | NA | NA | NA |
| *Bos grunniens* | NA | NA | NA | NA | NA | 25^[14]^ |
| *Bos javanicus birmanicus* | NA | NA | NA | NA | NA | NA |
| *Bos javanicus javanicus* | NA | NA | NA | NA | NA | 27^[14]^ |
| *Boselaphus tragocamelus* | NA | NA | NA | NA | NA | NA |
| *Bubalus bubalis bubalis* | NA | NA | NA | NA | NA | 25^[14]^ |
| *Bubalus bubalis carabanesis* | NA | NA | NA | NA | NA | 25^[14]^ |
| *Bubalus depressicornis* | NA | NA | NA | NA | NA | 24^[14]^ |
| *Budorcas taxicolor* | NA | NA | NA | NA | NA | 30^[12, 14]^ |
| *Capra caucasica* | NA | NA | NA | 49^[12]^ | NA | 34^[14]^ |
| *Capra falconeri* | NA | NA | NA | NA | NA | 35^[12, 14]^ |
| *Capra ibex* | NA | NA | NA | 54^[12]^ | NA | 30^[12, 14]^ |
| *Capra pyrenaica* | NA | NA | NA | NA | NA | NA |
| *Capra sibirica* | NA | NA | NA | NA | NA | NA |
| *Capricornis crispus* | NA | 6^[12]^ | NA | NA | NA | 31^[14]^ |
| *Cephalophus adersi* | NA | NA | NA | NA | NA | NA |
| *Cephalophus callipygus 1* | NA | NA | NA | NA | NA | NA |
| *Cephalophus callipygus 2* | NA | NA | NA | NA | NA | NA |
| *Cephalophus callipygus 3* | NA | NA | NA | NA | NA | NA |
| *Cephalophus dorsalis* | NA | NA | NA | NA | NA | 18^[14]^ |
| *Cephalophus jentinki* | NA | NA | NA | NA | NA | NA |
| *Cephalophus leucogaster* | NA | NA | NA | NA | NA | NA |
| *Cephalophus natalensis* | NA | NA | NA | NA | NA | NA |
| *Cephalophus nigrifrons* | NA | NA | NA | NA | NA | NA |
| *Cephalophus ogilbyi* | NA | NA | NA | NA | NA | NA |
| *Cephalophus rufilatus* | NA | NA | NA | NA | NA | NA |
| *Cephalophus silvicultor* | NA | NA | NA | NA | NA | NA |
| *Cephalophus spadix* | NA | NA | NA | NA | NA | NA |
| *Connochaetes gnou* | NA | NA | NA | 36^[12]^ | 16^[12]^ | 32^[12, 14]^ |
| *Connochaetes taurinus 1* | NA | NA | NA | 40^[12]^ | NA | 44^[12, 14]^ |
| *Connochaetes taurinus 2* | NA | NA | NA | 40^[12]^ | NA | 44^[12, 14]^ |
| *Damaliscus pygargus* | NA | NA | NA | NA | NA | 19^[14]^ |
| *Eudorcas rufifrons 1* | NA | NA | NA | NA | NA | NA |
| *Eudorcas rufifrons 2* | NA | NA | NA | NA | NA | NA |
| *Gazella bennettii* | NA | NA | NA | NA | NA | NA |
| *Gazella cuvieri* | NA | NA | NA | NA | 6^[12]^ | NA |
| *Gazella dorcas osiris* | NA | NA | NA | NA | 20^[12]^ | 21^[14]^ |
| *Gazella dorcas pelzelnii* | NA | NA | NA | NA | 20^[12]^ | 21^[14]^ |
| *Gazella gazella erlangeri* | NA | NA | NA | NA | NA | 16^[14]^ |
| *Gazella gazella gazella* | NA | NA | NA | NA | NA | 16^[14]^ |
| *Gazella leptoceros* | NA | NA | NA | NA | NA | NA |
| *Gazella spekei* | NA | NA | NA | NA | NA | 13^[14]^ |
| *Gazella subgutturosa marica* | NA | NA | NA | 30^[12]^ | NA | 16^[14]^ |
| *Gazella subgutturosa subgutturosa* | NA | NA | NA | 30^[12]^ | NA | 16^[14]^ |
| *Hemitragus jemlahicus* | NA | NA | NA | NA | NA | 59^[14]^ |
| *Hippotragus equinus* | NA | NA | NA | 36^[12]^ | NA | 28^[14]^ |
| *Hippotragus niger* | NA | NA | NA | 60^[12]^ | NA | 34^[12, 14]^ |
| *Kobus ellipsiprymnus* | NA | NA | NA | NA | NA | 46^[14]^ |
| *Kobus leche* | 54^[12]^ | NA | NA | NA | NA | 23^[12, 14]^ |
| *Litocranius walleri* | NA | NA | NA | NA | NA | 31^[14]^ |
| *Madoqua kirkii* | NA | NA | NA | NA | NA | 8^[14]^ |
| *Madoqua saltiana* | NA | NA | NA | NA | NA | 8^[12, 14]^ |
| *Nanger dama* | NA | NA | NA | NA | 4^[12]^ | 4^[14]^ |
| *Nanger granti* | NA | NA | NA | NA | NA | 8^[14]^ |
| *Nanger soemmerringii* | NA | NA | NA | NA | NA | 14^[14]^ |
| *Neotragus batesi* | NA | NA | NA | NA | NA | 11^[14]^ |
| *Neotragus moschatus* | NA | NA | NA | NA | NA | 6^[14]^ |
| *Oreamnos americanus* | 40^[12]^ | NA | 18^[12]^ | 21^[12]^ | NA | 28^[12, 14]^ |
| *Oreotragus oreotragus* | NA | NA | NA | NA | NA | 12^[12, 14]^ |
| *Oryx dammah* | NA | NA | NA | NA | NA | NA |
| *Oryx gazella* | NA | NA | NA | NA | NA | 28^[14]^ |
| *Ourebia ourebi* | NA | NA | NA | NA | NA | 13^[12, 14]^ |
| *Ovibos moschatus* | NA | NA | NA | NA | NA | 24^[12, 14]^ |
| *Pantholops hodgsonii* | NA | NA | NA | NA | NA | NA |
| *Pelea capreolus* | NA | NA | NA | NA | NA | NA |
| *Philantomba maxwelli* | NA | NA | NA | NA | NA | 11^[14]^ |
| *Philantomba monticola 1* | NA | NA | NA | 9^[12]^ | NA | 24^[14]^ |
| *Philantomba monticola 2* | NA | NA | NA | 9^[12]^ | NA | 24^[14]^ |
| *Procapra gutturosa* | NA | NA | NA | NA | NA | 30^[14]^ |
| *Pseudois nayaur* | NA | NA | NA | NA | NA | 14^[14]^ |
| *Pseudoryx nghetinhensis* | NA | NA | NA | NA | NA | 25^[14]^ |
| *Raphicerus campestris* | NA | NA | NA | NA | 9^[12]^ | 8^[12, 14]^ |
| *Redunca arundinum* | NA | NA | NA | NA | NA | NA |
| *Redunca fulvorufula* | NA | NA | 10^[12]^ | NA | NA | 11^[12, 14]^ |
| *Rupicapra pyrenaica* | NA | NA | NA | NA | NA | NA |
| *Rupicapra rupicapra* | NA | 18^[12]^ | NA | NA | 24^[12]^ | 24^[12, 14]^ |
| *Saiga tatarica* | NA | NA | NA | 22^[12]^ | NA | 11^[14]^ |
| *Sylvicapra grimmia* | NA | NA | NA | NA | NA | 8^[14]^ |
| *Syncerus caffer* | NA | NA | 33^[12]^ | 90^[12]^ | NA | 42^[12, 14]^ |
| *Tetracerus quadricornis* | NA | NA | NA | NA | NA | NA |
| *Tragelaphus angasii* | NA | 18^[12]^ | NA | 60^[12]^ | NA | 22^[14]^ |
| *Tragelaphus derbianus* | NA | NA | NA | NA | NA | 24^[14]^ |
| *Tragelaphus eurycerus* | NA | NA | NA | NA | NA | 30^[12, 14]^ |
| *Tragelaphus imberbis* | NA | NA | NA | NA | NA | 22^[14]^ |
| *Tragelaphus oryx* | NA | NA | NA | NA | NA | 21^[12, 14]^ |
| *Tragelaphus scriptus 1* | NA | 11^[12]^ | NA | NA | NA | 12^[12, 14]^ |
| *Tragelaphus scriptus 2* | NA | 11^[12]^ | NA | NA | NA | 12^[12, 14]^ |
| *Tragelaphus scriptus 3* | NA | 11^[12]^ | NA | NA | NA | 12^[12, 14]^ |
| *Tragelaphus spekii* | NA | NA | NA | NA | NA | 34^[12, 14]^ |
| *Tragelaphus strepsiceros* | NA | NA | NA | NA | NA | 22^[14]^ |
| *Giraffa camelopardalis* | NA | 42^[12]^ | NA | NA | NA | 54^[12, 14]^ |
| *Antilocapra americana* | NA | NA | NA | 17^[12]^ | NA | 28^[12]^ |

**Appendix S2: Female sexual maturity**

|  | Age (months after birth) at first: | | | | | | | |
| --- | --- | --- | --- | --- | --- | --- | --- | --- |
|  | Estrus | Mating | Conception | Pregnancy | Birth | Corpus Luteum | "Sexual Maturity" |  |
| *Alces alces* | NA | 24^[12]^ | NA | 12^[12]^ | 41^[12, 14]^ | 12^[12]^ | 22^[12, 14]^ |  |
| *Axis axis* | NA | NA | NA | NA | 15^[10]^ | NA | 17^[12, 14]^ |  |
| *Axis porcinus* | NA | NA | NA | NA | 10^[10]^ | NA | 14^[14]^ |  |
| *Blastocerus dichotomus* | NA | NA | NA | NA | NA | NA | NA |  |
| *Capreolus capreolus* | NA | 10^[12]^ | NA | NA | 24^[10, 12, 14, 17]^ | 8^[12]^ | 15^[12, 14]^ |  |
| *Cervus elaphus* | NA | 33^[12]^ | 23^[12]^ | 17^[12]^ | 33^[12, 14]^ | NA | 19^[12, 14]^ |  |
| *Cervus nippon centralis* | NA | 42^[12]^ | NA | 10^[12]^ | 36^[12, 14]^ | NA | 17^[12, 14]^ |  |
| *Cervus nippon taiouanus* | NA | 42^[12]^ | NA | 10^[12]^ | 36^[12, 14]^ | NA | 17^[12, 14]^ |  |
| *Cervus nippon yakushimae* | NA | 42^[12]^ | NA | 10^[12]^ | 36^[12, 14]^ | NA | 17^[12, 14]^ |  |
| *Dama dama dama* | NA | 36^[12]^ | NA | NA | 36^[12, 14]^ | NA | 18^[12, 14]^ |  |
| *Dama dama mesopotamica* | NA | NA | NA | NA | NA | NA | NA |  |
| *Elaphodus cephalophus* | NA | NA | NA | NA | NA | NA | 20^[12, 14]^ |  |
| *Elaphurus davidianus* | NA | NA | NA | NA | NA | NA | NA |  |
| *Hydropotes inermis* | NA | NA | NA | 6^[12]^ | 8^[10]^ | NA | 10^[14]^ |  |
| *Mazama gouazoubira* | NA | NA | NA | NA | NA | NA | 21^[10, 11, 13]^ |  |
| *Mazama rufina* | NA | NA | NA | NA | NA | NA | 12^[9]^ |  |
| *Muntiacus crinifrons* | NA | NA | NA | NA | NA | NA | NA |  |
| *Muntiacus muntjak* | NA | NA | NA | NA | NA | NA | 10^[12, 14]^ |  |
| *Muntiacus reevesi* | NA | NA | NA | NA | 6^[10]^ | NA | 8^[12, 14]^ |  |
| *Muntiacus vuquangensis* | NA | NA | NA | NA | NA | NA | NA |  |
| *Odocoileus hemionus* | NA | 18^[12]^ | 7^[12]^ | NA | 17^[12, 14]^ | 6^[12]^ | 16^[12, 14]^ |  |
| *Odocoileus virginianus 1* | 11^[12]^ | 10^[12]^ | 13^[12]^ | 11^[9, 12]^ | 15^[12, 14]^ | 7^[12]^ | 10^[12, 14]^ |  |
| *Odocoileus virginianus 2* | 11^[12]^ | 10^[12]^ | 13^[12]^ | 11^[9, 12]^ | 15^[12, 14]^ | 7^[12]^ | 10^[12, 14]^ |  |
| *Odocoileus virginianus 3* | 11^[12]^ | 10^[12]^ | 13^[12]^ | 11^[9, 12]^ | 15^[12, 14]^ | 7^[12]^ | 10^[12, 14]^ |  |
| *Ozotoceros bezoarcticus* | NA | NA | NA | NA | 24^[9]^ | NA | 13^[9, 12, 14]^ |  |
| *Przewalskium albirostris* | NA | NA | NA | NA | NA | NA | 24^[16]^ |  |
| *Pudu mephistophiles* | NA | NA | NA | NA | NA | NA | 13^[9]^ |  |
| *Pudu puda* | NA | 6^[12]^ | NA | NA | NA | NA | 7^[9, 14]^ |  |
| *Rangifer tarandus* | NA | 27^[12]^ | 11^[12]^ | 21^[12]^ | 29^[10]^ | NA | 27^[12, 14]^ |  |
| *Rucervus duvauceli* | NA | NA | NA | NA | 36^[9, 13]^ | NA | 28^[14]^ |  |
| *Rucervus eldi* | NA | NA | 16^[12]^ | NA | 32^[12, 14]^ | NA | 18^[14]^ |  |
| *Rusa timorensis* | NA | 12^[12]^ | 27^[12]^ | NA | NA | NA | 21^[12, 14]^ |  |
| *Rusa unicolor* | NA | NA | NA | NA | 27^[12, 14]^ | NA | 19^[14]^ |  |
| *Moschus moschiferus* | NA | NA | NA | NA | NA | NA | 13^[12, 14]^ |  |
| *Addax nasomaculatus* | NA | NA | NA | NA | 35^[12, 14]^ | NA | NA |  |
| *Aepyceros melampus* | NA | 15^[12]^ | 18^[12]^ | NA | 24^[12, 14]^ | NA | 17^[14]^ |  |
| *Alcelaphus buselaphus buselaphus* | NA | 24^[12]^ | NA | NA | 31^[12, 14]^ | NA | 28^[14]^ |  |
| *Alcelaphus buselaphus lichtensteinii* | NA | NA | NA | NA | 28^[14]^ | NA | 21^[14]^ |  |
| *Ammotragus lervia* | NA | NA | NA | NA | 13^[12, 14]^ | NA | 17^[14]^ |  |
| *Antidorcas marsupialis* | NA | NA | 11^[12]^ | NA | NA | NA | 11^[14]^ |  |
| *Antilope cervicapra* | 7^[12]^ | NA | NA | NA | 22^[12, 14]^ | NA | 23^[12, 14]^ |  |
| *Arabitragus jayakari* | NA | NA | NA | NA | NA | NA | NA |  |
| *Bison bison* | NA | 28^[12]^ | 30^[12]^ | 42^[12]^ | 36^[12, 14]^ | NA | 27^[12, 14]^ |  |
| *Bison bonasus* | NA | NA | NA | NA | 36^[12, 14]^ | NA | 29^[12, 14]^ |  |
| *Bos gaurus* | NA | NA | NA | NA | NA | NA | NA |  |
| *Bos grunniens* | NA | NA | NA | NA | NA | NA | 25^[14]^ |  |
| *Bos javanicus birmanicus* | NA | NA | NA | NA | 30^[12, 14]^ | NA | 25^[12, 14]^ |  |
| *Bos javanicus javanicus* | NA | NA | NA | NA | 30^[12, 14]^ | NA | 25^[12, 14]^ |  |
| *Boselaphus tragocamelus* | NA | NA | NA | NA | 33^[12, 14]^ | NA | NA |  |
| *Bubalus bubalis bubalis* | NA | NA | 29^[12]^ | NA | NA | 14^[12]^ | 25^[14]^ |  |
| *Bubalus bubalis carabanesis* | NA | NA | 29^[12]^ | NA | NA | 14^[12]^ | 25^[14]^ |  |
| *Bubalus depressicornis* | NA | NA | NA | NA | NA | NA | 24^[14]^ |  |
| *Budorcas taxicolor* | NA | NA | NA | NA | NA | NA | 30^[12, 14]^ |  |
| *Capra caucasica* | 24^[12]^ | NA | NA | NA | NA | NA | 34^[14]^ |  |
| *Capra falconeri* | 30^[12]^ | NA | NA | NA | 24^[12, 14]^ | NA | 29^[12, 14]^ |  |
| *Capra ibex* | NA | 42^[12]^ | NA | NA | 25^[12, 14]^ | NA | 30^[12, 14]^ |  |
| *Capra pyrenaica* | NA | NA | NA | NA | NA | NA | NA |  |
| *Capra sibirica* | NA | NA | NA | NA | NA | NA | NA |  |
| *Capricornis crispus* | NA | NA | NA | NA | NA | NA | 31^[14]^ |  |
| *Cephalophus adersi* | NA | NA | NA | NA | NA | NA | NA |  |
| *Cephalophus callipygus 1* | NA | NA | NA | NA | NA | NA | NA |  |
| *Cephalophus callipygus 2* | NA | NA | NA | NA | NA | NA | NA |  |
| *Cephalophus callipygus 3* | NA | NA | NA | NA | NA | NA | NA |  |
| *Cephalophus dorsalis* | NA | NA | 14^[12]^ | NA | 25^[12, 14]^ | NA | 18^[14]^ |  |
| *Cephalophus jentinki* | NA | NA | NA | NA | NA | NA | NA |  |
| *Cephalophus leucogaster* | NA | NA | NA | NA | NA | NA | NA |  |
| *Cephalophus natalensis* | NA | NA | NA | NA | NA | NA | NA |  |
| *Cephalophus nigrifrons* | NA | NA | NA | NA | NA | NA | NA |  |
| *Cephalophus ogilbyi* | NA | NA | NA | NA | NA | NA | NA |  |
| *Cephalophus rufilatus* | NA | NA | NA | NA | 26^[12, 14]^ | NA | NA |  |
| *Cephalophus silvicultor* | NA | NA | NA | NA | NA | NA | NA |  |
| *Cephalophus spadix* | NA | NA | NA | NA | NA | NA | NA |  |
| *Connochaetes gnou* | NA | 36^[12]^ | 28^[12]^ | NA | 33^[12, 14]^ | NA | 25^[12, 14]^ |  |
| *Connochaetes taurinus 1* | NA | 14^[12]^ | 30^[12]^ | NA | 24^[12, 14]^ | NA | 26^[12, 14]^ |  |
| *Connochaetes taurinus 2* | NA | 14^[12]^ | 30^[12]^ | NA | 24^[12, 14]^ | NA | 26^[12, 14]^ |  |
| *Damaliscus pygargus* | NA | NA | NA | NA | 33^[14]^ | NA | 19^[14]^ |  |
| *Eudorcas rufifrons 1* | NA | NA | NA | NA | NA | NA | NA |  |
| *Eudorcas rufifrons 2* | NA | NA | NA | NA | NA | NA | NA |  |
| *Gazella bennettii* | NA | NA | NA | NA | NA | NA | NA |  |
| *Gazella cuvieri* | NA | NA | NA | NA | 11^[12]^ | NA | NA |  |
| *Gazella dorcas osiris* | NA | NA | 21^[12]^ | NA | 28^[12, 14]^ | NA | 21^[14]^ |  |
| *Gazella dorcas pelzelnii* | NA | NA | 21^[12]^ | NA | 28^[12, 14]^ | NA | 21^[14]^ |  |
| *Gazella gazella erlangeri* | NA | NA | 12^[12]^ | NA | 18^[12, 14]^ | NA | 17^[12, 14]^ |  |
| *Gazella gazella gazella* | NA | NA | 12^[12]^ | NA | 18^[12, 14]^ | NA | 17^[12, 14]^ |  |
| *Gazella leptoceros* | NA | NA | NA | NA | 11^[12, 14]^ | NA | NA |  |
| *Gazella spekei* | NA | NA | 17^[12]^ | NA | NA | NA | 13^[14]^ |  |
| *Gazella subgutturosa marica* | NA | 19^[12]^ | NA | NA | NA | NA | 14^[14]^ |  |
| *Gazella subgutturosa subgutturosa* | NA | 19^[12]^ | NA | NA | NA | NA | 14^[14]^ |  |
| *Hemitragus jemlahicus* | NA | NA | NA | NA | 24^[12, 14]^ | NA | 51^[14]^ |  |
| *Hippotragus equinus* | NA | NA | 24^[12]^ | NA | 35^[12, 14]^ | NA | 26^[12, 14]^ |  |
| *Hippotragus niger* | 27^[12]^ | NA | NA | NA | 35^[12, 14]^ | NA | 32^[12, 14]^ |  |
| *Kobus ellipsiprymnus* | NA | NA | NA | NA | NA | NA | 46^[14]^ |  |
| *Kobus leche* | NA | 30^[12]^ | NA | 36^[12]^ | 21^[12, 14]^ | NA | 31^[14]^ |  |
| *Litocranius walleri* | NA | NA | NA | NA | 20^[12, 14]^ | NA | 27^[12, 14]^ |  |
| *Madoqua kirkii* | NA | NA | NA | NA | 17^[12, 14]^ | NA | 8^[12, 14]^ |  |
| *Madoqua saltiana* | NA | NA | NA | NA | NA | NA | 8^[12, 14]^ |  |
| *Nanger dama* | NA | NA | NA | NA | NA | NA | 4^[14]^ |  |
| *Nanger granti* | NA | 11^[12]^ | 5^[12]^ | NA | NA | NA | 8^[14]^ |  |
| *Nanger soemmerringii* | NA | NA | 19^[12]^ | NA | 27^[14]^ | NA | 14^[14]^ |  |
| *Neotragus batesi* | NA | NA | NA | NA | 12^[12, 14]^ | NA | 11^[14]^ |  |
| *Neotragus moschatus* | NA | NA | NA | NA | NA | NA | 6^[12, 14]^ |  |
| *Oreamnos americanus* | NA | 30^[12]^ | NA | 34^[12]^ | 36^[12, 14]^ | 27^[12]^ | 28^[12, 14]^ |  |
| *Oreotragus oreotragus* | NA | NA | NA | NA | NA | NA | 12^[12, 14]^ |  |
| *Oryx dammah* | NA | NA | NA | NA | 33^[12, 14]^ | NA | NA |  |
| *Oryx gazella* | NA | NA | NA | NA | 29^[12, 14]^ | NA | 28^[12, 14]^ |  |
| *Ourebia ourebi* | NA | NA | NA | NA | 22^[12, 14]^ | NA | 11^[12, 14]^ |  |
| *Ovibos moschatus* | 15^[12]^ | 36^[12]^ | NA | NA | 45^[12, 14]^ | NA | 23^[12, 14]^ |  |
| *Pantholops hodgsonii* | NA | NA | NA | NA | NA | NA | NA |  |
| *Pelea capreolus* | NA | NA | NA | NA | NA | NA | NA |  |
| *Philantomba maxwelli* | NA | NA | NA | NA | 36^[12, 14]^ | NA | 11^[14]^ |  |
| *Philantomba monticola 1* | NA | 12^[12]^ | NA | NA | 19^[14]^ | NA | 24^[12, 14]^ |  |
| *Philantomba monticola 2* | NA | 12^[12]^ | NA | NA | 19^[14]^ | NA | 24^[12, 14]^ |  |
| *Procapra gutturosa* | NA | NA | NA | NA | NA | NA | 30^[14]^ |  |
| *Pseudois nayaur* | NA | NA | NA | NA | 24^[12, 14]^ | NA | 13^[14]^ |  |
| *Pseudoryx nghetinhensis* | NA | NA | NA | NA | NA | NA | 25^[14]^ |  |
| *Raphicerus campestris* | NA | NA | 10^[12]^ | NA | NA | NA | 7^[12, 14]^ |  |
| *Redunca arundinum* | NA | NA | NA | NA | NA | NA | NA |  |
| *Redunca fulvorufula* | NA | NA | NA | NA | NA | NA | 13^[12, 14]^ |  |
| *Rupicapra pyrenaica* | NA | NA | NA | NA | 24^[14]^ | NA | NA |  |
| *Rupicapra rupicapra* | NA | 27^[12]^ | NA | 22^[12]^ | 37^[12, 14]^ | NA | 27^[12, 14]^ |  |
| *Saiga tatarica* | NA | 12^[12]^ | NA | NA | NA | NA | 9^[12, 14]^ |  |
| *Sylvicapra grimmia* | NA | 12^[12]^ | 9^[12]^ | NA | 20^[14]^ | NA | 10^[14]^ |  |
| *Syncerus caffer* | NA | NA | NA | 59^[12]^ | 48^[12, 14]^ | 60^[12]^ | 40^[12, 14]^ |  |
| *Tetracerus quadricornis* | NA | NA | NA | NA | 17^[12, 14]^ | NA | NA |  |
| *Tragelaphus angasii* | NA | NA | NA | NA | 23^[12, 14]^ | NA | 21^[14]^ |  |
| *Tragelaphus derbianus* | NA | NA | NA | NA | NA | NA | 24^[14]^ |  |
| *Tragelaphus eurycerus* | NA | NA | 29^[12]^ | NA | NA | NA | 27^[12, 14]^ |  |
| *Tragelaphus imberbis* | NA | NA | NA | NA | 37^[12, 14]^ | NA | 22^[14]^ |  |
| *Tragelaphus oryx* | 20^[12]^ | 29^[12]^ | 13^[12]^ | NA | 35^[12]^ | NA | 22^[14]^ |  |
| *Tragelaphus scriptus 1* | NA | NA | 18^[12]^ | NA | 21^[12, 14]^ | NA | 14^[12, 14]^ |  |
| *Tragelaphus scriptus 2* | NA | NA | 18^[12]^ | NA | 21^[12, 14]^ | NA | 14^[12, 14]^ |  |
| *Tragelaphus scriptus 3* | NA | NA | 18^[12]^ | NA | 21^[12, 14]^ | NA | 14^[12, 14]^ |  |
| *Tragelaphus spekii* | NA | NA | NA | NA | 21^[12, 14]^ | NA | 34^[12, 14]^ |  |
| *Tragelaphus strepsiceros* | NA | 17^[12]^ | NA | 17^[12]^ | 30^[12, 14]^ | NA | 22^[14]^ |  |
| *Giraffa camelopardalis* | NA | 56^[12]^ | 56^[12]^ | 63^[12]^ | 61^[14]^ | NA | 47^[12, 14]^ |  |
| *Antilocapra americana* | NA | 17^[12]^ | 5^[7]^ | 8^[12]^ | 12^[12]^ | NA | 14^[12]^ |  |

**Shape and size data**

All shape data are from Caro et al. (2003) except those for *Moschus moschiferus*, *Giraffa camelopardalis*, and *Antilocapra americana*, which were encoded by the author.

**Appendix S3: Male shape data**

|  | Shape | | | | | | | | | | | | |
| --- | --- | --- | --- | --- | --- | --- | --- | --- | --- | --- | --- | --- | --- |
|  | Tip above boss | Tip below boss | Tip inront of boss | Tip behind boss | Tip on side of boss | Tip in line with boss | Smooth | Crenulated | Straight | Twisted | Simple spike | Two to five tines | Greater than 5 tines |
| *Alces alces* | 1 | 0 | 0 | 1 | 1 | 0 | 1 | 0 | 1 | 0 | 0 | 0 | 1 |
| *Axis axis* | 1 | 0 | 0 | 1 | 1 | 0 | 1 | 0 | 1 | 0 | 0 | 1 | 0 |
| *Axis porcinus* | 1 | 0 | 0 | 1 | 1 | 0 | 1 | 0 | 1 | 0 | 0 | 1 | 0 |
| *Blastocerus dichotomus* | 1 | 0 | 0 | 1 | 1 | 0 | 1 | 0 | 1 | 0 | 0 | 1 | 0 |
| *Capreolus capreolus* | 1 | 0 | 0 | 1 | 0 | 1 | 1 | 0 | 1 | 0 | 0 | 1 | 0 |
| *Cervus elaphus* | 1 | 0 | 0 | 1 | 1 | 0 | 1 | 0 | 1 | 0 | 0 | 1 | 0 |
| *Cervus nippon centralis* | 1 | 0 | 0 | 1 | 1 | 0 | 1 | 0 | 1 | 0 | 0 | 1 | 0 |
| *Cervus nippon taiouanus* | 1 | 0 | 0 | 1 | 1 | 0 | 1 | 0 | 1 | 0 | 0 | 1 | 0 |
| *Cervus nippon yakushimae* | 1 | 0 | 0 | 1 | 1 | 0 | 1 | 0 | 1 | 0 | 0 | 1 | 0 |
| *Dama dama dama* | 1 | 0 | 0 | 1 | 1 | 0 | 1 | 0 | 1 | 0 | 0 | 0 | 1 |
| *Dama dama mesopotamica* | 1 | 0 | 0 | 1 | 1 | 0 | 1 | 0 | 1 | 0 | 0 | 0 | 1 |
| *Elaphodus cephalophus* | 1 | 0 | 0 | 0 | 0 | 1 | 1 | 0 | 1 | 0 | 1 | 0 | 0 |
| *Elaphurus davidianus* | NA | NA | NA | NA | NA | NA | NA | NA | NA | NA | NA | NA | NA |
| *Hydropotes inermis* | 0 | 0 | 0 | 0 | 0 | 0 | 0 | 0 | 0 | 0 | 0 | 0 | 0 |
| *Mazama gouazoubira* | NA | NA | NA | NA | NA | NA | NA | NA | NA | NA | NA | NA | NA |
| *Mazama rufina* | NA | NA | NA | NA | NA | NA | NA | NA | NA | NA | NA | NA | NA |
| *Muntiacus crinifrons* | 1 | 0 | 0 | 1 | 0 | 1 | 1 | 0 | 1 | 0 | 1 | 0 | 0 |
| *Muntiacus muntjak* | 1 | 0 | 0 | 1 | 0 | 1 | 1 | 0 | 1 | 0 | 1 | 0 | 0 |
| *Muntiacus reevesi* | 1 | 0 | 0 | 1 | 0 | 1 | 1 | 0 | 1 | 0 | 1 | 0 | 0 |
| *Muntiacus vuquangensis* | 1 | 0 | 0 | 1 | 0 | 1 | 1 | 0 | 1 | 0 | 1 | 0 | 0 |
| *Odocoileus hemionus* | 1 | 0 | 0 | 1 | 0 | 1 | 1 | 0 | 1 | 0 | 0 | 1 | 0 |
| *Odocoileus virginianus 1* | 1 | 0 | 0 | 1 | 1 | 0 | 1 | 0 | 1 | 0 | 0 | 1 | 0 |
| *Odocoileus virginianus 2* | 1 | 0 | 0 | 1 | 1 | 0 | 1 | 0 | 1 | 0 | 0 | 1 | 0 |
| *Odocoileus virginianus 3* | 1 | 0 | 0 | 1 | 1 | 0 | 1 | 0 | 1 | 0 | 0 | 1 | 0 |
| *Ozotoceros bezoarcticus* | 1 | 0 | 0 | 1 | 0 | 1 | 1 | 0 | 1 | 0 | 0 | 1 | 0 |
| *Przewalskium albirostris* | 1 | 0 | 0 | 1 | 1 | 0 | 1 | 0 | 1 | 0 | 0 | 1 | 0 |
| *Pudu mephistophiles* | NA | NA | NA | NA | NA | NA | NA | NA | NA | NA | NA | NA | NA |
| *Pudu puda* | 1 | 0 | 0 | 1 | 0 | 1 | 1 | 0 | 1 | 0 | 1 | 0 | 0 |
| *Rangifer tarandus* | 1 | 0 | 0 | 1 | 1 | 0 | 1 | 0 | 1 | 0 | 0 | 0 | 1 |
| *Rucervus duvauceli* | 1 | 0 | 0 | 1 | 1 | 0 | 1 | 0 | 1 | 0 | 0 | 0 | 1 |
| *Rucervus eldi* | 1 | 0 | 0 | 1 | 1 | 0 | 1 | 0 | 1 | 0 | 0 | 1 | 0 |
| *Rusa timorensis* | 1 | 0 | 0 | 1 | 1 | 0 | 1 | 0 | 1 | 0 | 0 | 1 | 0 |
| *Rusa unicolor* | 1 | 0 | 0 | 1 | 1 | 0 | 1 | 0 | 1 | 0 | 0 | 1 | 0 |
| *Moschus moschiferus* | 0 | 0 | 0 | 0 | 0 | 0 | 0 | 0 | 0 | 0 | 0 | 0 | 0 |
| *Addax nasomaculatus* | 1 | 0 | 0 | 1 | 0 | 1 | 0 | 1 | 0 | 1 | 1 | 0 | 0 |
| *Aepyceros melampus* | 1 | 0 | 0 | 1 | 0 | 1 | 0 | 1 | 1 | 0 | 1 | 0 | 0 |
| *Alcelaphus buselaphus buselaphus* | 1 | 0 | 0 | 1 | 1 | 0 | 0 | 1 | 0 | 1 | 1 | 0 | 0 |
| *Alcelaphus buselaphus lichtensteinii* | 1 | 0 | 0 | 1 | 0 | 1 | 0 | 1 | 0 | 1 | 1 | 0 | 0 |
| *Ammotragus lervia* | 0 | 1 | 0 | 1 | 1 | 0 | 0 | 1 | 0 | 1 | 1 | 0 | 0 |
| *Antidorcas marsupialis* | 1 | 0 | 1 | 0 | 0 | 1 | 0 | 1 | 1 | 0 | 1 | 0 | 0 |
| *Antilope cervicapra* | 1 | 0 | 0 | 1 | 1 | 0 | 0 | 1 | 0 | 1 | 1 | 0 | 0 |
| *Arabitragus jayakari* | 0 | 1 | 0 | 1 | 1 | 0 | 1 | 0 | 0 | 1 | 1 | 0 | 0 |
| *Bison bison* | 1 | 0 | 0 | 1 | 1 | 0 | 1 | 0 | 1 | 0 | 1 | 0 | 0 |
| *Bison bonasus* | NA | NA | NA | NA | NA | NA | NA | NA | NA | NA | NA | NA | NA |
| *Bos gaurus* | 1 | 0 | 0 | 1 | 1 | 0 | 1 | 0 | 1 | 0 | 1 | 0 | 0 |
| *Bos grunniens* | 1 | 0 | 1 | 0 | 1 | 0 | 1 | 0 | 1 | 0 | 1 | 0 | 0 |
| *Bos javanicus birmanicus* | 1 | 0 | 1 | 0 | 1 | 0 | 1 | 0 | 1 | 0 | 1 | 0 | 0 |
| *Bos javanicus javanicus* | 1 | 0 | 1 | 0 | 1 | 0 | 1 | 0 | 1 | 0 | 1 | 0 | 0 |
| *Boselaphus tragocamelus* | 1 | 0 | 0 | 1 | 0 | 1 | 1 | 0 | 1 | 0 | 1 | 0 | 0 |
| *Bubalus bubalis bubalis* | NA | NA | NA | NA | NA | NA | NA | NA | NA | NA | NA | NA | NA |
| *Bubalus bubalis carabanesis* | NA | NA | NA | NA | NA | NA | NA | NA | NA | NA | NA | NA | NA |
| *Bubalus depressicornis* | 1 | 0 | 0 | 1 | 0 | 1 | 0 | 1 | 1 | 0 | 1 | 0 | 0 |
| *Budorcas taxicolor* | 1 | 0 | 0 | 1 | 1 | 0 | 0 | 1 | 0 | 1 | 1 | 0 | 0 |
| *Capra caucasica* | 0 | 1 | 0 | 1 | 1 | 0 | 0 | 1 | 0 | 1 | 1 | 0 | 0 |
| *Capra falconeri* | 1 | 0 | 0 | 1 | 1 | 0 | 1 | 0 | 0 | 1 | 1 | 0 | 0 |
| *Capra ibex* | 0 | 1 | 0 | 1 | 1 | 0 | 0 | 1 | 0 | 1 | 1 | 0 | 0 |
| *Capra pyrenaica* | 0 | 1 | 0 | 1 | 1 | 0 | 1 | 0 | 0 | 1 | 1 | 0 | 0 |
| *Capra sibirica* | NA | NA | NA | NA | NA | NA | NA | NA | NA | NA | NA | NA | NA |
| *Capricornis crispus* | 1 | 0 | 0 | 1 | 0 | 1 | 0 | 1 | 0 | 1 | 1 | 0 | 0 |
| *Cephalophus adersi* | 1 | 0 | 0 | 1 | 0 | 1 | 0 | 1 | 1 | 0 | 1 | 0 | 0 |
| *Cephalophus callipygus 1* | 1 | 0 | 0 | 1 | 0 | 1 | 0 | 1 | 1 | 0 | 1 | 0 | 0 |
| *Cephalophus callipygus 2* | 1 | 0 | 0 | 1 | 0 | 1 | 0 | 1 | 1 | 0 | 1 | 0 | 0 |
| *Cephalophus callipygus 3* | 1 | 0 | 0 | 1 | 0 | 1 | 0 | 1 | 1 | 0 | 1 | 0 | 0 |
| *Cephalophus dorsalis* | 1 | 0 | 0 | 1 | 0 | 1 | 0 | 1 | 1 | 0 | 1 | 0 | 0 |
| *Cephalophus jentinki* | 1 | 0 | 0 | 1 | 0 | 1 | 0 | 1 | 1 | 0 | 1 | 0 | 0 |
| *Cephalophus leucogaster* | 1 | 0 | 0 | 1 | 0 | 1 | 0 | 1 | 1 | 0 | 1 | 0 | 0 |
| *Cephalophus natalensis* | 1 | 0 | 0 | 1 | 0 | 1 | 0 | 1 | 1 | 0 | 1 | 0 | 0 |
| *Cephalophus nigrifrons* | 1 | 0 | 0 | 1 | 0 | 1 | 0 | 1 | 1 | 0 | 1 | 0 | 0 |
| *Cephalophus ogilbyi* | 1 | 0 | 0 | 1 | 0 | 1 | 0 | 1 | 1 | 0 | 1 | 0 | 0 |
| *Cephalophus rufilatus* | 1 | 0 | 0 | 1 | 0 | 1 | 0 | 1 | 1 | 0 | 1 | 0 | 0 |
| *Cephalophus silvicultor* | 1 | 0 | 0 | 1 | 0 | 1 | 0 | 1 | 1 | 0 | 1 | 0 | 0 |
| *Cephalophus spadix* | 1 | 0 | 0 | 1 | 0 | 1 | 0 | 1 | 1 | 0 | 1 | 0 | 0 |
| *Connochaetes gnou* | 1 | 0 | 1 | 0 | 1 | 0 | 0 | 1 | 0 | 1 | 1 | 0 | 0 |
| *Connochaetes taurinus 1* | 1 | 0 | 0 | 1 | 1 | 0 | 0 | 1 | 0 | 1 | 1 | 0 | 0 |
| *Connochaetes taurinus 2* | 1 | 0 | 0 | 1 | 1 | 0 | 0 | 1 | 0 | 1 | 1 | 0 | 0 |
| *Damaliscus pygargus* | 1 | 0 | 0 | 1 | 1 | 0 | 0 | 1 | 0 | 1 | 1 | 0 | 0 |
| *Eudorcas rufifrons 1* | 1 | 0 | 0 | 1 | 0 | 1 | 0 | 1 | 1 | 0 | 1 | 0 | 0 |
| *Eudorcas rufifrons 2* | 1 | 0 | 0 | 1 | 0 | 1 | 0 | 1 | 1 | 0 | 1 | 0 | 0 |
| *Gazella bennettii* | 1 | 0 | 0 | 1 | 0 | 1 | 0 | 1 | 1 | 0 | 1 | 0 | 0 |
| *Gazella cuvieri* | 1 | 0 | 0 | 1 | 0 | 1 | 0 | 1 | 1 | 0 | 1 | 0 | 0 |
| *Gazella dorcas osiris* | 1 | 0 | 0 | 1 | 0 | 1 | 0 | 1 | 1 | 0 | 1 | 0 | 0 |
| *Gazella dorcas pelzelnii* | 1 | 0 | 0 | 1 | 0 | 1 | 0 | 1 | 1 | 0 | 1 | 0 | 0 |
| *Gazella gazella erlangeri* | 1 | 0 | 0 | 1 | 0 | 1 | 0 | 1 | 1 | 0 | 1 | 0 | 0 |
| *Gazella gazella gazella* | 1 | 0 | 0 | 1 | 0 | 1 | 0 | 1 | 1 | 0 | 1 | 0 | 0 |
| *Gazella leptoceros* | 1 | 0 | 0 | 1 | 0 | 1 | 0 | 1 | 1 | 0 | 1 | 0 | 0 |
| *Gazella spekei* | 1 | 0 | 0 | 1 | 0 | 1 | 0 | 1 | 1 | 0 | 1 | 0 | 0 |
| *Gazella subgutturosa marica* | 1 | 0 | 0 | 1 | 0 | 1 | 0 | 1 | 1 | 0 | 1 | 0 | 0 |
| *Gazella subgutturosa subgutturosa* | 1 | 0 | 0 | 1 | 0 | 1 | 0 | 1 | 1 | 0 | 1 | 0 | 0 |
| *Hemitragus jemlahicus* | 0 | 1 | 0 | 1 | 1 | 0 | 1 | 0 | 0 | 1 | 1 | 0 | 0 |
| *Hippotragus equinus* | 1 | 0 | 0 | 1 | 0 | 1 | 0 | 1 | 1 | 0 | 1 | 0 | 0 |
| *Hippotragus niger* | 1 | 0 | 0 | 1 | 0 | 1 | 0 | 1 | 1 | 0 | 1 | 0 | 0 |
| *Kobus ellipsiprymnus* | 1 | 0 | 0 | 1 | 0 | 1 | 0 | 1 | 1 | 0 | 1 | 0 | 0 |
| *Kobus leche* | 1 | 0 | 0 | 1 | 0 | 1 | 0 | 1 | 1 | 0 | 1 | 0 | 0 |
| *Litocranius walleri* | 1 | 0 | 0 | 1 | 0 | 1 | 0 | 1 | 1 | 0 | 1 | 0 | 0 |
| *Madoqua kirkii* | 1 | 0 | 0 | 1 | 0 | 1 | 0 | 1 | 1 | 0 | 1 | 0 | 0 |
| *Madoqua saltiana* | NA | NA | NA | NA | NA | NA | NA | NA | NA | NA | NA | NA | NA |
| *Nanger dama* | 1 | 0 | 0 | 1 | 0 | 1 | 0 | 1 | 1 | 0 | 1 | 0 | 0 |
| *Nanger granti* | 1 | 0 | 0 | 1 | 1 | 0 | 0 | 1 | 1 | 0 | 1 | 0 | 0 |
| *Nanger soemmerringii* | 1 | 0 | 0 | 1 | 0 | 1 | 0 | 1 | 1 | 0 | 1 | 0 | 0 |
| *Neotragus batesi* | NA | NA | NA | NA | NA | NA | NA | NA | NA | NA | NA | NA | NA |
| *Neotragus moschatus* | 1 | 0 | 0 | 1 | 0 | 1 | 0 | 1 | 1 | 0 | 1 | 0 | 0 |
| *Oreamnos americanus* | 1 | 0 | 0 | 1 | 0 | 1 | 1 | 0 | 1 | 0 | 1 | 0 | 0 |
| *Oreotragus oreotragus* | 1 | 0 | 0 | 1 | 0 | 1 | 0 | 1 | 1 | 0 | 1 | 0 | 0 |
| *Oryx dammah* | 1 | 0 | 0 | 1 | 0 | 1 | 0 | 1 | 1 | 0 | 1 | 0 | 0 |
| *Oryx gazella* | 1 | 0 | 0 | 1 | 0 | 1 | 0 | 1 | 1 | 0 | 1 | 0 | 0 |
| *Ourebia ourebi* | 1 | 0 | 0 | 1 | 0 | 1 | 0 | 1 | 1 | 0 | 1 | 0 | 0 |
| *Ovibos moschatus* | 0 | 1 | 1 | 0 | 1 | 0 | 1 | 0 | 0 | 1 | 1 | 0 | 0 |
| *Pantholops hodgsonii* | 1 | 0 | 1 | 0 | 0 | 1 | 0 | 1 | 1 | 0 | 1 | 0 | 0 |
| *Pelea capreolus* | 1 | 0 | 0 | 1 | 0 | 1 | 1 | 0 | 1 | 0 | 1 | 0 | 0 |
| *Philantomba maxwelli* | 1 | 0 | 0 | 1 | 0 | 1 | 0 | 1 | 1 | 0 | 1 | 0 | 0 |
| *Philantomba monticola 1* | 1 | 0 | 0 | 1 | 0 | 1 | 0 | 1 | 1 | 0 | 1 | 0 | 0 |
| *Philantomba monticola 2* | 1 | 0 | 0 | 1 | 0 | 1 | 0 | 1 | 1 | 0 | 1 | 0 | 0 |
| *Procapra gutturosa* | 1 | 0 | 0 | 1 | 0 | 1 | 0 | 1 | 1 | 0 | 1 | 0 | 0 |
| *Pseudois nayaur* | 0 | 1 | 0 | 1 | 1 | 0 | 1 | 0 | 0 | 1 | 1 | 0 | 0 |
| *Pseudoryx nghetinhensis* | 1 | 0 | 0 | 1 | 0 | 1 | 1 | 0 | 1 | 0 | 1 | 0 | 0 |
| *Raphicerus campestris* | 1 | 0 | 0 | 1 | 0 | 1 | 0 | 1 | 1 | 0 | 1 | 0 | 0 |
| *Redunca arundinum* | 1 | 0 | 1 | 0 | 0 | 1 | 0 | 1 | 1 | 0 | 1 | 0 | 0 |
| *Redunca fulvorufula* | 1 | 0 | 1 | 0 | 0 | 1 | 0 | 1 | 1 | 0 | 1 | 0 | 0 |
| *Rupicapra pyrenaica* | 1 | 0 | 0 | 1 | 0 | 1 | 1 | 0 | 0 | 1 | 1 | 0 | 0 |
| *Rupicapra rupicapra* | 1 | 0 | 0 | 1 | 0 | 1 | 1 | 0 | 0 | 1 | 1 | 0 | 0 |
| *Saiga tatarica* | 1 | 0 | 0 | 1 | 0 | 1 | 0 | 1 | 1 | 0 | 1 | 0 | 0 |
| *Sylvicapra grimmia* | 1 | 0 | 0 | 1 | 0 | 1 | 0 | 1 | 1 | 0 | 1 | 0 | 0 |
| *Syncerus caffer* | 1 | 0 | 0 | 1 | 1 | 0 | 1 | 0 | 1 | 0 | 1 | 0 | 0 |
| *Tetracerus quadricornis* | 1 | 0 | 0 | 1 | 0 | 1 | 1 | 0 | 1 | 0 | 1 | 0 | 0 |
| *Tragelaphus angasii* | 1 | 0 | 0 | 1 | 1 | 0 | 1 | 0 | 0 | 1 | 1 | 0 | 0 |
| *Tragelaphus derbianus* | 1 | 0 | 0 | 1 | 0 | 1 | 1 | 0 | 0 | 1 | 1 | 0 | 0 |
| *Tragelaphus eurycerus* | 1 | 0 | 0 | 1 | 0 | 1 | 1 | 0 | 0 | 1 | 1 | 0 | 0 |
| *Tragelaphus imberbis* | 1 | 0 | 0 | 1 | 0 | 1 | 1 | 0 | 0 | 1 | 1 | 0 | 0 |
| *Tragelaphus oryx* | NA | NA | NA | NA | NA | NA | NA | NA | NA | NA | NA | NA | NA |
| *Tragelaphus scriptus 1* | 1 | 0 | 0 | 1 | 0 | 1 | 1 | 0 | 0 | 1 | 1 | 0 | 0 |
| *Tragelaphus scriptus 2* | 1 | 0 | 0 | 1 | 0 | 1 | 1 | 0 | 0 | 1 | 1 | 0 | 0 |
| *Tragelaphus scriptus 3* | 1 | 0 | 0 | 1 | 0 | 1 | 1 | 0 | 0 | 1 | 1 | 0 | 0 |
| *Tragelaphus spekii* | 1 | 0 | 0 | 1 | 0 | 1 | 1 | 0 | 0 | 1 | 1 | 0 | 0 |
| *Tragelaphus strepsiceros* | 1 | 0 | 0 | 1 | 0 | 1 | 1 | 0 | 0 | 1 | 1 | 0 | 0 |
| *Giraffa camelopardalis* | 1 | 0 | 0 | 1 | 0 | 1 | 1 | 0 | 1 | 0 | 1 | 0 | 0 |
| *Antilocapra americana* | 1 | 0 | 1 | 1 | 1 | 1 | 1 | 0 | 0 | 1 | 0 | 1 | 0 |

**Appendix S4: Female shape data**

|  | Shape | | | | | | | | | | | | | |  |
| --- | --- | --- | --- | --- | --- | --- | --- | --- | --- | --- | --- | --- | --- | --- | --- |
|  | | Tip above boss | Tip below boss | Tip in front of boss | Tip behind boss | Tip on side of boss | Tip in line with boss | Smooth | Crenulated | Straight | Twisted | Simple spike | Two to five tines | Greater than 5 tines | |
| *Alces alces* | | 0 | 0 | 0 | 0 | 0 | 0 | 0 | 0 | 0 | 0 | 0 | 0 | 0 | |
| *Axis axis* | | 0 | 0 | 0 | 0 | 0 | 0 | 0 | 0 | 0 | 0 | 0 | 0 | 0 | |
| *Axis porcinus* | | 0 | 0 | 0 | 0 | 0 | 0 | 0 | 0 | 0 | 0 | 0 | 0 | 0 | |
| *Blastocerus dichotomus* | | 0 | 0 | 0 | 0 | 0 | 0 | 0 | 0 | 0 | 0 | 0 | 0 | 0 | |
| *Capreolus capreolus* | | 0 | 0 | 0 | 0 | 0 | 0 | 0 | 0 | 0 | 0 | 0 | 0 | 0 | |
| *Cervus elaphus* | | 0 | 0 | 0 | 0 | 0 | 0 | 0 | 0 | 0 | 0 | 0 | 0 | 0 | |
| *Cervus nippon centralis* | | 0 | 0 | 0 | 0 | 0 | 0 | 0 | 0 | 0 | 0 | 0 | 0 | 0 | |
| *Cervus nippon taiouanus* | | 0 | 0 | 0 | 0 | 0 | 0 | 0 | 0 | 0 | 0 | 0 | 0 | 0 | |
| *Cervus nippon yakushimae* | | 0 | 0 | 0 | 0 | 0 | 0 | 0 | 0 | 0 | 0 | 0 | 0 | 0 | |
| *Dama dama dama* | | 0 | 0 | 0 | 0 | 0 | 0 | 0 | 0 | 0 | 0 | 0 | 0 | 0 | |
| *Dama dama mesopotamica* | | 0 | 0 | 0 | 0 | 0 | 0 | 0 | 0 | 0 | 0 | 0 | 0 | 0 | |
| *Elaphodus cephalophus* | | 0 | 0 | 0 | 0 | 0 | 0 | 0 | 0 | 0 | 0 | 0 | 0 | 0 | |
| *Elaphurus davidianus* | | NA | NA | NA | NA | NA | NA | NA | NA | NA | NA | NA | NA | NA | |
| *Hydropotes inermis* | | 0 | 0 | 0 | 0 | 0 | 0 | 0 | 0 | 0 | 0 | 0 | 0 | 0 | |
| *Mazama gouazoubira* | | NA | NA | NA | NA | NA | NA | NA | NA | NA | NA | NA | NA | NA | |
| *Mazama rufina* | | NA | NA | NA | NA | NA | NA | NA | NA | NA | NA | NA | NA | NA | |
| *Muntiacus crinifrons* | | 0 | 0 | 0 | 0 | 0 | 0 | 0 | 0 | 0 | 0 | 0 | 0 | 0 | |
| *Muntiacus muntjak* | | 0 | 0 | 0 | 0 | 0 | 0 | 0 | 0 | 0 | 0 | 0 | 0 | 0 | |
| *Muntiacus reevesi* | | 0 | 0 | 0 | 0 | 0 | 0 | 0 | 0 | 0 | 0 | 0 | 0 | 0 | |
| *Muntiacus vuquangensis* | | 0 | 0 | 0 | 0 | 0 | 0 | 0 | 0 | 0 | 0 | 0 | 0 | 0 | |
| *Odocoileus hemionus* | | 0 | 0 | 0 | 0 | 0 | 0 | 0 | 0 | 0 | 0 | 0 | 0 | 0 | |
| *Odocoileus virginianus 1* | | 0 | 0 | 0 | 0 | 0 | 0 | 0 | 0 | 0 | 0 | 0 | 0 | 0 | |
| *Odocoileus virginianus 2* | | 0 | 0 | 0 | 0 | 0 | 0 | 0 | 0 | 0 | 0 | 0 | 0 | 0 | |
| *Odocoileus virginianus 3* | | 0 | 0 | 0 | 0 | 0 | 0 | 0 | 0 | 0 | 0 | 0 | 0 | 0 | |
| *Ozotoceros bezoarcticus* | | 0 | 0 | 0 | 0 | 0 | 0 | 0 | 0 | 0 | 0 | 0 | 0 | 0 | |
| *Przewalskium albirostris* | | 0 | 0 | 0 | 0 | 0 | 0 | 0 | 0 | 0 | 0 | 0 | 0 | 0 | |
| *Pudu mephistophiles* | | NA | NA | NA | NA | NA | NA | NA | NA | NA | NA | NA | NA | NA | |
| *Pudu puda* | | 0 | 0 | 0 | 0 | 0 | 0 | 0 | 0 | 0 | 0 | 0 | 0 | 0 | |
| *Rangifer tarandus* | | 1 | 0 | 0 | 1 | 1 | 0 | 1 | 0 | 1 | 0 | 0 | 0 | 1 | |
| *Rucervus duvauceli* | | 0 | 0 | 0 | 0 | 0 | 0 | 0 | 0 | 0 | 0 | 0 | 0 | 0 | |
| *Rucervus eldi* | | 0 | 0 | 0 | 0 | 0 | 0 | 0 | 0 | 0 | 0 | 0 | 0 | 0 | |
| *Rusa timorensis* | | 0 | 0 | 0 | 0 | 0 | 0 | 0 | 0 | 0 | 0 | 0 | 0 | 0 | |
| *Rusa unicolor* | | 0 | 0 | 0 | 0 | 0 | 0 | 0 | 0 | 0 | 0 | 0 | 0 | 0 | |
| *Moschus moschiferus* | | 0 | 0 | 0 | 0 | 0 | 0 | 0 | 0 | 0 | 0 | 0 | 0 | 0 | |
| *Addax nasomaculatus* | | 1 | 0 | 0 | 1 | 0 | 1 | 0 | 1 | 0 | 1 | 1 | 0 | 0 | |
| *Aepyceros melampus* | | 0 | 0 | 0 | 0 | 0 | 0 | 0 | 0 | 0 | 0 | 0 | 0 | 0 | |
| *Alcelaphus buselaphus buselaphus* | | 1 | 0 | 0 | 1 | 1 | 0 | 0 | 1 | 0 | 1 | 1 | 0 | 0 | |
| *Alcelaphus buselaphus lichtensteinii* | | 1 | 0 | 0 | 1 | 0 | 1 | 0 | 1 | 0 | 1 | 1 | 0 | 0 | |
| *Ammotragus lervia* | | 0 | 1 | 0 | 1 | 1 | 0 | 0 | 1 | 0 | 1 | 1 | 0 | 0 | |
| *Antidorcas marsupialis* | | 1 | 0 | 1 | 0 | 0 | 1 | 0 | 1 | 1 | 0 | 1 | 0 | 0 | |
| *Antilope cervicapra* | | 0 | 0 | 0 | 0 | 0 | 0 | 0 | 0 | 0 | 0 | 0 | 0 | 0 | |
| *Arabitragus jayakari* | | 0 | 1 | 0 | 1 | 1 | 0 | 1 | 0 | 0 | 1 | 1 | 0 | 0 | |
| *Bison bison* | | 1 | 0 | 0 | 1 | 1 | 0 | 1 | 0 | 1 | 0 | 1 | 0 | 0 | |
| *Bison bonasus* | | NA | NA | NA | NA | NA | NA | NA | NA | NA | NA | NA | NA | NA | |
| *Bos gaurus* | | 1 | 0 | 0 | 1 | 1 | 0 | 1 | 0 | 1 | 0 | 1 | 0 | 0 | |
| *Bos grunniens* | | 1 | 0 | 1 | 0 | 1 | 0 | 1 | 0 | 1 | 0 | NA | NA | NA | |
| *Bos javanicus birmanicus* | | 1 | 0 | 1 | 0 | 1 | 0 | 1 | 0 | 1 | 0 | 1 | 0 | 0 | |
| *Bos javanicus javanicus* | | 1 | 0 | 1 | 0 | 1 | 0 | 1 | 0 | 1 | 0 | NA | NA | NA | |
| *Boselaphus tragocamelus* | | 0 | 0 | 0 | 0 | 0 | 0 | 0 | 0 | 0 | 0 | 0 | 0 | 0 | |
| *Bubalus bubalis bubalis* | | NA | NA | NA | NA | NA | NA | NA | NA | NA | NA | NA | NA | NA | |
| *Bubalus bubalis carabanesis* | | NA | NA | NA | NA | NA | NA | NA | NA | NA | NA | NA | NA | NA | |
| *Bubalus depressicornis* | | 1 | 0 | 0 | 1 | 0 | 1 | 0 | 1 | 1 | 0 | 1 | 0 | 0 | |
| *Budorcas taxicolor* | | 1 | 0 | 0 | 1 | 1 | 0 | 0 | 1 | 0 | 1 | 1 | 0 | 0 | |
| *Capra caucasica* | | 0 | 1 | 0 | 1 | 1 | 0 | 0 | 1 | 0 | 1 | 1 | 0 | 0 | |
| *Capra falconeri* | | 1 | 0 | 0 | 1 | 1 | 0 | 1 | 0 | 0 | 1 | 1 | 0 | 0 | |
| *Capra ibex* | | 0 | 1 | 0 | 1 | 1 | 0 | 0 | 1 | 0 | 1 | 1 | 0 | 0 | |
| *Capra pyrenaica* | | 0 | 1 | 0 | 1 | 1 | 0 | 1 | 0 | 0 | 1 | 1 | 0 | 0 | |
| *Capra sibirica* | | NA | NA | NA | NA | NA | NA | NA | NA | NA | NA | NA | NA | NA | |
| *Capricornis crispus* | | 1 | 0 | 0 | 1 | 0 | 1 | 0 | 1 | 0 | 1 | 1 | 0 | 0 | |
| *Cephalophus adersi* | | 1 | 0 | 0 | 1 | 0 | 1 | 0 | 1 | 1 | 0 | 1 | 0 | 0 | |
| *Cephalophus callipygus 1* | | 1 | 0 | 0 | 1 | 0 | 1 | 0 | 1 | 1 | 0 | 1 | 0 | 0 | |
| *Cephalophus callipygus 2* | | 1 | 0 | 0 | 1 | 0 | 1 | 0 | 1 | 1 | 0 | 1 | 0 | 0 | |
| *Cephalophus callipygus 3* | | 1 | 0 | 0 | 1 | 0 | 1 | 0 | 1 | 1 | 0 | 1 | 0 | 0 | |
| *Cephalophus dorsalis* | | 1 | 0 | 0 | 1 | 0 | 1 | 0 | 1 | 1 | 0 | 1 | 0 | 0 | |
| *Cephalophus jentinki* | | 1 | 0 | 0 | 1 | 0 | 1 | 0 | 1 | 1 | 0 | 1 | 0 | 0 | |
| *Cephalophus leucogaster* | | 1 | 0 | 0 | 1 | 0 | 1 | 0 | 1 | 1 | 0 | 1 | 0 | 0 | |
| *Cephalophus natalensis* | | 1 | 0 | 0 | 1 | 0 | 1 | 0 | 1 | 1 | 0 | 1 | 0 | 0 | |
| *Cephalophus nigrifrons* | | 1 | 0 | 0 | 1 | 0 | 1 | 0 | 1 | 1 | 0 | 1 | 0 | 0 | |
| *Cephalophus ogilbyi* | | 1 | 0 | 0 | 1 | 0 | 1 | 0 | 1 | 1 | 0 | 1 | 0 | 0 | |
| *Cephalophus rufilatus* | | 1 | 0 | 0 | 1 | 0 | 1 | 0 | 1 | 1 | 0 | 1 | 0 | 0 | |
| *Cephalophus silvicultor* | | 1 | 0 | 0 | 1 | 0 | 1 | 0 | 1 | 1 | 0 | 1 | 0 | 0 | |
| *Cephalophus spadix* | | 1 | 0 | 0 | 1 | 0 | 1 | 0 | 1 | 1 | 0 | 1 | 0 | 0 | |
| *Connochaetes gnou* | | 1 | 0 | 1 | 0 | 1 | 0 | 0 | 1 | 0 | 1 | 1 | 0 | 0 | |
| *Connochaetes taurinus 1* | | 1 | 0 | 0 | 1 | 1 | 0 | 0 | 1 | 0 | 1 | 1 | 0 | 0 | |
| *Connochaetes taurinus 2* | | 1 | 0 | 0 | 1 | 1 | 0 | 0 | 1 | 0 | 1 | 1 | 0 | 0 | |
| *Damaliscus pygargus* | | 1 | 0 | 0 | 1 | 1 | 0 | 0 | 1 | 0 | 1 | 1 | 0 | 0 | |
| *Eudorcas rufifrons 1* | | 1 | 0 | 0 | 1 | 0 | 1 | 0 | 1 | 1 | 0 | 1 | 0 | 0 | |
| *Eudorcas rufifrons 2* | | 1 | 0 | 0 | 1 | 0 | 1 | 0 | 1 | 1 | 0 | 1 | 0 | 0 | |
| *Gazella bennettii* | | 1 | 0 | 0 | 1 | 0 | 1 | 0 | 1 | 1 | 0 | 1 | 0 | 0 | |
| *Gazella cuvieri* | | 1 | 0 | 0 | 1 | 0 | 1 | 0 | 1 | 1 | 0 | 1 | 0 | 0 | |
| *Gazella dorcas osiris* | | 1 | 0 | 0 | 1 | 0 | 1 | 0 | 1 | 1 | 0 | 1 | 0 | 0 | |
| *Gazella dorcas pelzelnii* | | 1 | 0 | 0 | 1 | 0 | 1 | 0 | 1 | 1 | 0 | 1 | 0 | 0 | |
| *Gazella gazella erlangeri* | | 1 | 0 | 0 | 1 | 0 | 1 | 0 | 1 | 1 | 0 | 1 | 0 | 0 | |
| *Gazella gazella gazella* | | 1 | 0 | 0 | 1 | 0 | 1 | 0 | 1 | 1 | 0 | 1 | 0 | 0 | |
| *Gazella leptoceros* | | 1 | 0 | 0 | 1 | 0 | 1 | 0 | 1 | 1 | 0 | 1 | 0 | 0 | |
| *Gazella spekei* | | 1 | 0 | 0 | 1 | 0 | 1 | 0 | 1 | 1 | 0 | 1 | 0 | 0 | |
| *Gazella subgutturosa marica* | | 0 | 0 | 0 | 0 | 0 | 0 | 0 | 0 | 0 | 0 | 0 | 0 | 0 | |
| *Gazella subgutturosa subgutturosa* | | 0 | 0 | 0 | 0 | 0 | 0 | 0 | 0 | 0 | 0 | 0 | 0 | 0 | |
| *Hemitragus jemlahicus* | | 0 | 1 | 0 | 1 | 1 | 0 | 1 | 0 | 0 | 1 | 1 | 0 | 0 | |
| *Hippotragus equinus* | | 1 | 0 | 0 | 1 | 0 | 1 | 0 | 1 | 1 | 0 | 1 | 0 | 0 | |
| *Hippotragus niger* | | 1 | 0 | 0 | 1 | 0 | 1 | 0 | 1 | 1 | 0 | 1 | 0 | 0 | |
| *Kobus ellipsiprymnus* | | 0 | 0 | 0 | 0 | 0 | 0 | 0 | 0 | 0 | 0 | 0 | 0 | 0 | |
| *Kobus leche* | | 0 | 0 | 0 | 0 | 0 | 0 | 0 | 0 | 0 | 0 | 0 | 0 | 0 | |
| *Litocranius walleri* | | 0 | 0 | 0 | 0 | 0 | 0 | 0 | 0 | 0 | 0 | 0 | 0 | 0 | |
| *Madoqua kirkii* | | 0 | 0 | 0 | 0 | 0 | 0 | 0 | 0 | 0 | 0 | 0 | 0 | 0 | |
| *Madoqua saltiana* | | NA | NA | NA | NA | NA | NA | NA | NA | NA | NA | NA | NA | NA | |
| *Nanger dama* | | 1 | 0 | 0 | 1 | 0 | 1 | 0 | 1 | 1 | 0 | 1 | 0 | 0 | |
| *Nanger granti* | | 1 | 0 | 0 | 1 | 1 | 0 | 0 | 1 | 1 | 0 | 1 | 0 | 0 | |
| *Nanger soemmerringii* | | 1 | 0 | 0 | 1 | 0 | 1 | 0 | 1 | 1 | 0 | 1 | 0 | 0 | |
| *Neotragus batesi* | | NA | NA | NA | NA | NA | NA | NA | NA | NA | NA | NA | NA | NA | |
| *Neotragus moschatus* | | 0 | 0 | 0 | 0 | 0 | 0 | 0 | 0 | 0 | 0 | NA | NA | NA | |
| *Oreamnos americanus* | | 1 | 0 | 0 | 1 | 0 | 1 | 1 | 0 | 1 | 0 | 1 | 0 | 0 | |
| *Oreotragus oreotragus* | | 0 | 0 | 0 | 0 | 0 | 0 | 0 | 0 | 0 | 0 | 0 | 0 | 0 | |
| *Oryx dammah* | | 1 | 0 | 0 | 1 | 0 | 1 | 0 | 1 | 1 | 0 | 1 | 0 | 0 | |
| *Oryx gazella* | | 1 | 0 | 0 | 1 | 0 | 1 | 0 | 1 | 1 | 0 | 1 | 0 | 0 | |
| *Ourebia ourebi* | | 0 | 0 | 0 | 0 | 0 | 0 | 0 | 0 | 0 | 0 | 0 | 0 | 0 | |
| *Ovibos moschatus* | | 0 | 1 | 1 | 0 | 1 | 0 | 1 | 0 | 0 | 1 | 1 | 0 | 0 | |
| *Pantholops hodgsonii* | | 0 | 0 | 0 | 0 | 0 | 0 | 0 | 0 | 0 | 0 | 0 | 0 | 0 | |
| *Pelea capreolus* | | 0 | 0 | 0 | 0 | 0 | 0 | 0 | 0 | 0 | 0 | 0 | 0 | 0 | |
| *Philantomba maxwelli* | | 1 | 0 | 0 | 1 | 0 | 1 | 0 | 1 | 1 | 0 | 1 | 0 | 0 | |
| *Philantomba monticola 1* | | 1 | 0 | 0 | 1 | 0 | 1 | 0 | 1 | 1 | 0 | 1 | 0 | 0 | |
| *Philantomba monticola 2* | | 1 | 0 | 0 | 1 | 0 | 1 | 0 | 1 | 1 | 0 | 1 | 0 | 0 | |
| *Procapra gutturosa* | | 0 | 0 | 0 | 0 | 0 | 0 | 0 | 0 | 0 | 0 | 0 | 0 | 0 | |
| *Pseudois nayaur* | | 0 | 1 | 0 | 1 | 1 | 0 | 1 | 0 | 0 | 1 | 1 | 0 | 0 | |
| *Pseudoryx nghetinhensis* | | 1 | 0 | 0 | 1 | 0 | 1 | 1 | 0 | 1 | 0 | 1 | 0 | 0 | |
| *Raphicerus campestris* | | 0 | 0 | 0 | 0 | 0 | 0 | 0 | 0 | 0 | 0 | 0 | 0 | 0 | |
| *Redunca arundinum* | | 0 | 0 | 0 | 0 | 0 | 0 | 0 | 0 | 0 | 0 | 0 | 0 | 0 | |
| *Redunca fulvorufula* | | 0 | 0 | 0 | 0 | 0 | 0 | 0 | 0 | 0 | 0 | 0 | 0 | 0 | |
| *Rupicapra pyrenaica* | | 1 | 0 | 0 | 1 | 0 | 1 | 1 | 0 | 0 | 1 | 1 | 0 | 0 | |
| *Rupicapra rupicapra* | | 1 | 0 | 0 | 1 | 0 | 1 | 1 | 0 | 0 | 1 | 1 | 0 | 0 | |
| *Saiga tatarica* | | 0 | 0 | 0 | 0 | 0 | 0 | 0 | 0 | 0 | 0 | 0 | 0 | 0 | |
| *Sylvicapra grimmia* | | 0 | 0 | 0 | 0 | 0 | 0 | 0 | 0 | 0 | 0 | 0 | 0 | 0 | |
| *Syncerus caffer* | | 1 | 0 | 0 | 1 | 1 | 0 | 1 | 0 | 1 | 0 | 1 | 0 | 0 | |
| *Tetracerus quadricornis* | | 0 | 0 | 0 | 0 | 0 | 0 | 0 | 0 | 0 | 0 | 0 | 0 | 0 | |
| *Tragelaphus angasii* | | 0 | 0 | 0 | 0 | 0 | 0 | 0 | 0 | 0 | 0 | 0 | 0 | 0 | |
| *Tragelaphus derbianus* | | 1 | 0 | 0 | 1 | 0 | 1 | 1 | 0 | 0 | 1 | 1 | 0 | 0 | |
| *Tragelaphus eurycerus* | | 1 | 0 | 0 | 1 | 0 | 1 | 1 | 0 | 0 | 1 | 1 | 0 | 0 | |
| *Tragelaphus imberbis* | | 0 | 0 | 0 | 0 | 0 | 0 | 0 | 0 | 0 | 0 | 0 | 0 | 0 | |
| *Tragelaphus oryx* | | NA | NA | NA | NA | NA | NA | NA | NA | NA | NA | NA | NA | NA | |
| *Tragelaphus scriptus 1* | | 0 | 0 | 0 | 0 | 0 | 0 | 0 | 0 | 0 | 0 | 0 | 0 | 0 | |
| *Tragelaphus scriptus 2* | | 0 | 0 | 0 | 0 | 0 | 0 | 0 | 0 | 0 | 0 | 0 | 0 | 0 | |
| *Tragelaphus scriptus 3* | | 0 | 0 | 0 | 0 | 0 | 0 | 0 | 0 | 0 | 0 | 0 | 0 | 0 | |
| *Tragelaphus spekii* | | 0 | 0 | 0 | 0 | 0 | 0 | 0 | 0 | 0 | 0 | 0 | 0 | 0 | |
| *Tragelaphus strepsiceros* | | 0 | 0 | 0 | 0 | 0 | 0 | 0 | 0 | 0 | 0 | 0 | 0 | 0 | |
| *Giraffa camelopardalis* | | 1 | 0 | 0 | 1 | 0 | 1 | 1 | 0 | 1 | 0 | 1 | 0 | 0 | |
| *Antilocapra americana* | | 1 | 0 | 1 | 1 | 1 | 1 | 1 | 0 | 0 | 1 | 0 | 1 | 0 | |

**Appendix S5: Body mass and cranial appendage length**

|  | Body mass (kg) | | Cranial appendage length (cm) | |
| --- | --- | --- | --- | --- |
|  | Male | Female | Male | Female |
| *Alces alces* | 482.5^[15]^ | 324.5^[13, 18]^ | 144^[15]^ | 0 |
| *Axis axis* | 89.5^[15]^ | 48.3^[11, 15]^ | 84.5^[15]^ | 0 |
| *Axis porcinus* | 41^[15]^ | 35^[13, 15]^ | 39.9^[15]^ | 0 |
| *Blastocerus dichotomus* | 130^[15]^ | 120^[13, 15]^ | 60^[15]^ | 0 |
| *Capreolus capreolus* | 28^[15]^ | 26.0^[13, 15, 19]^ | 23.4^[15]^ | 0 |
| *Cervus elaphus* | 259^[1, 15]^ | 168.6^[1, 15, 19]^ | 133.7^[15]^ | 0 |
| *Cervus nippon centralis* | 52^[15]^ | 41^[13, 15]^ | 48^[15]^ | 0 |
| *Cervus nippon taiouanus* | 52^[15]^ | 41^[13, 15]^ | 48^[15]^ | 0 |
| *Cervus nippon yakushimae* | 52^[15]^ | 41^[13, 15]^ | 48^[15]^ | 0 |
| *Dama dama dama* | 67^[15]^ | 44^[13, 15]^ | 61.5^[15]^ | 0 |
| *Dama dama mesopotamica* | 67^[15]^ | 44^[13, 15]^ | 61.5^[15]^ | 0 |
| *Elaphodus cephalophus* | 18^[15]^ | 18^[6, 15]^ | 2.5^[15]^ | 0 |
| *Elaphurus davidianus* | 214^[15]^ | 149^[11, 15]^ | 73.7^[15]^ | 0 |
| *Hydropotes inermis* | 12^[13]^ | 9.5^[13]^ | 0 | 0 |
| *Mazama gouazoubira* | NA | NA | NA | NA |
| *Mazama rufina* | NA | NA | NA | NA |
| *Muntiacus crinifrons* | 23^[15]^ | 24.1^[15]^ | 3.6^[15]^ | 0 |
| *Muntiacus muntjak* | 19^[15]^ | 18^[11, 15]^ | 14.2^[15]^ | 0 |
| *Muntiacus reevesi* | 13.5^[13, 15]^ | 12^[13, 15, 19]^ | 11.4^[15]^ | 0 |
| *Muntiacus vuquangensis* | 45^[15]^ | 34^[15]^ | 22.8^[15]^ | 0 |
| *Odocoileus hemionus* | 112.5^[13, 15]^ | 56.5^[13, 15, 19]^ | 88.5^[15]^ | 0 |
| *Odocoileus virginianus 1* | 154.5^[13, 15]^ | 45^[13, 15, 19]^ | 65.6^[15]^ | 0 |
| *Odocoileus virginianus 2* | 154.5^[13, 15]^ | 45^[13, 15, 19]^ | 65.6^[15]^ | 0 |
| *Odocoileus virginianus 3* | 154.5^[13, 15]^ | 45^[13, 15, 19]^ | 65.6^[15]^ | 0 |
| *Ozotoceros bezoarcticus* | 33.5^[13, 15]^ | 35^[13, 15]^ | 21^[15]^ | 0 |
| *Przewalskium albirostris* | 204^[15]^ | 190^[11]^ | 115^[15]^ | 0 |
| *Pudu mephistophiles* | NA | NA | NA | NA |
| *Pudu puda* | 13.3^[15, 21]^ | 13.5^[15, 21]^ | 8.5^[15]^ | 0 |
| *Rangifer tarandus* | 139.8^[1, 15]^ | 89.1^[1, 11, 13, 15, 19]^ | 91^[15]^ | 36.5^[3, 18]^ |
| *Rucervus duvauceli* | 236^[15]^ | 145^[11]^ | 81.3^[15]^ | 0 |
| *Rucervus eldi* | 105^[15]^ | 80^[11, 15]^ | 97.2^[15]^ | 0 |
| *Rusa timorensis* | 95.5^[15]^ | 107.0^[11, 15]^ | 67.5^[15]^ | 0 |
| *Rusa unicolor* | 192^[15]^ | 270^[11, 15]^ | 104.9^[15]^ | 0 |
| *Moschus moschiferus* | 15^[6]^ | 12^[6]^ | 0 | 0 |
| *Addax nasomaculatus* | 117.7^[4]^ | 84.7^[4]^ | 84.5^[4]^ | 67.5^[4]^ |
| *Aepyceros melampus* | 56.9^[4]^ | 43.8^[4]^ | 68.4^[4]^ | 0^[4]^ |
| *Alcelaphus buselaphus buselaphus* | NA | NA | NA | NA |
| *Alcelaphus buselaphus lichtensteinii* | 178.3^[4]^ | 162.7^[4]^ | 53.5^[4]^ | 50^[4]^ |
| *Ammotragus lervia* | 111.8^[4]^ | 51.6^[4]^ | 73.6^[4]^ | 37.8^[4]^ |
| *Antidorcas marsupialis* | 40.7^[4]^ | 35.5^[4]^ | 26.5^[4]^ | 19.6^[4]^ |
| *Antilope cervicapra* | 40.2^[4]^ | 34.4^[4]^ | 57^[4]^ | 0^[4]^ |
| *Arabitragus jayakari* | NA | NA | NA | NA |
| *Bison bison* | NA | NA | NA | NA |
| *Bison bonasus* | NA | NA | NA | NA |
| *Bos gaurus* | NA | NA | NA | NA |
| *Bos grunniens* | 590.5^[4]^ | 306^[4]^ | 80^[4]^ | 51^[4]^ |
| *Bos javanicus birmanicus* | NA | NA | NA | NA |
| *Bos javanicus javanicus* | NA | NA | NA | NA |
| *Boselaphus tragocamelus* | 253.3^[4]^ | 136.3^[4]^ | 19.5^[4]^ | 0 |
| *Bubalus bubalis bubalis* | 1200^[4]^ | 800^[4]^ | 105^[4]^ | 120^[4]^ |
| *Bubalus bubalis carabanesis* | 1200^[4]^ | 800^[4]^ | 105^[4]^ | 120^[4]^ |
| *Bubalus depressicornis* | 156^[4]^ | 145^[4]^ | 27.8^[4]^ | 22^[4]^ |
| *Budorcas taxicolor* | 282.7^[4]^ | 160^[4]^ | 41.2^[4]^ | 37^[4]^ |
| *Capra caucasica* | 86.3^[4]^ | 55^[4]^ | 83^[4]^ | 22.5^[4]^ |
| *Capra falconeri* | 92.7^[4]^ | 36.3^[4]^ | 108^[4]^ | 25^[4]^ |
| *Capra ibex* | 80.5^[4]^ | 48.9^[4]^ | 90^[4]^ | 26.5^[4]^ |
| *Capra pyrenaica* | 72.5^[4]^ | 40^[4]^ | 74^[4]^ | 22.5^[4]^ |
| *Capra sibirica* | 90^[4]^ | 44.2^[4]^ | 115.1^[4]^ | 27.3^[4]^ |
| *Capricornis crispus* | NA | NA | NA | NA |
| *Cephalophus adersi* | 9.3^[4]^ | 9.8^[4]^ | 4.5^[4]^ | 2.8^[4]^ |
| *Cephalophus callipygus 1* | 18.6^[4]^ | 18.4^[4]^ | 8^[4]^ | 4.9^[4]^ |
| *Cephalophus callipygus 2* | 18.6^[4]^ | 18.4^[4]^ | 8^[4]^ | 4.9^[4]^ |
| *Cephalophus callipygus 3* | 18.6^[4]^ | 18.4^[4]^ | 8^[4]^ | 4.9^[4]^ |
| *Cephalophus dorsalis* | 20.3^[4]^ | 19.5^[4]^ | 7^[4]^ | 8^[4]^ |
| *Cephalophus jentinki* | NA | NA | NA | NA |
| *Cephalophus leucogaster* | NA | NA | NA | NA |
| *Cephalophus natalensis* | 13.1^[4]^ | 12.1^[4]^ | 7^[4]^ | 3.5^[4]^ |
| *Cephalophus nigrifrons* | 13.3^[4]^ | 15^[4]^ | 10^[4]^ | 6^[4]^ |
| *Cephalophus ogilbyi* | 20.4^[4]^ | 22.5^[4]^ | 10^[4]^ | 4^[4]^ |
| *Cephalophus rufilatus* | 10.1^[4]^ | 10.3^[4]^ | 7^[4]^ | 3.5^[4]^ |
| *Cephalophus silvicultor* | NA | NA | NA | NA |
| *Cephalophus spadix* | NA | NA | NA | NA |
| *Connochaetes gnou* | 166.7^[4]^ | 135^[4]^ | 64^[4]^ | 52.5^[4]^ |
| *Connochaetes taurinus 1* | 235.3^[4]^ | 184.9^[4]^ | 68^[4]^ | 35^[4]^ |
| *Connochaetes taurinus 2* | 235.3^[4]^ | 184.9^[4]^ | 68^[4]^ | 35^[4]^ |
| *Damaliscus pygargus* | NA | NA | NA | NA |
| *Eudorcas rufifrons 1* | 27^[4]^ | 20.7^[4]^ | 40.4^[4]^ | 39.3^[4]^ |
| *Eudorcas rufifrons 2* | 27^[4]^ | 20.7^[4]^ | 40.4^[4]^ | 39.3^[4]^ |
| *Gazella bennettii* | NA | NA | NA | NA |
| *Gazella cuvieri* | 29.4^[4]^ | 20.4^[4]^ | 31^[4]^ | 25^[4]^ |
| *Gazella dorcas osiris* | 16.3^[4]^ | 13.3^[4]^ | 31.5^[4]^ | 20^[4]^ |
| *Gazella dorcas pelzelnii* | 16.3^[4]^ | 13.3^[4]^ | 31.5^[4]^ | 20^[4]^ |
| *Gazella gazella erlangeri* | 23.3^[4]^ | 20.8^[4]^ | 31^[4]^ | 11^[4]^ |
| *Gazella gazella gazella* | 23.3^[4]^ | 20.8^[4]^ | 31^[4]^ | 11^[4]^ |
| *Gazella leptoceros* | 27.2^[4]^ | 20.9^[4]^ | 36^[4]^ | 24^[4]^ |
| *Gazella spekei* | 21.3^[4]^ | 16.6^[4]^ | 28^[4]^ | 20^[4]^ |
| *Gazella subgutturosa marica* | 27.4^[4]^ | 23.2^[4]^ | 30^[4]^ | 0^[4]^ |
| *Gazella subgutturosa subgutturosa* | 27.4^[4]^ | 23.2^[4]^ | 30^[4]^ | 0^[4]^ |
| *Hemitragus jemlahicus* | 103.3^[4]^ | 56^[4]^ | 29.5^[4]^ | 19.3^[4]^ |
| *Hippotragus equinus* | 274.4^[4]^ | 256.4^[4]^ | 85^[4]^ | 70^[4]^ |
| *Hippotragus niger* | 235.2^[4]^ | 216.6^[4]^ | 122.5^[4]^ | 80^[4]^ |
| *Kobus ellipsiprymnus* | 236.8^[4]^ | 187.3^[4]^ | 75^[4]^ | 0^[4]^ |
| *Kobus leche* | 104.3^[4]^ | 78.7^[4]^ | 70^[4]^ | 0^[4]^ |
| *Litocranius walleri* | 35^[4]^ | 34.3^[4]^ | 38^[4]^ | 0^[4]^ |
| *Madoqua kirkii* | 4.6^[4]^ | 5.1^[4]^ | 7.5^[4]^ | 0^[4]^ |
| *Madoqua saltiana* | 2.3^[4]^ | 2.6^[4]^ | 5^[4]^ | 0^[4]^ |
| *Nanger dama* | NA | NA | NA | NA |
| *Nanger granti* | 72.1^[4]^ | 46^[4]^ | 65^[4]^ | 37.5^[4]^ |
| *Nanger soemmerringii* | NA | NA | NA | NA |
| *Neotragus batesi* | 2.4^[4]^ | 2.8^[4]^ | 3^[4]^ | 0^[4]^ |
| *Neotragus moschatus* | 4.8^[4]^ | 5.1^[4]^ | 9^[4]^ | 0^[4]^ |
| *Oreamnos americanus* | 95.9^[4]^ | 61^[4]^ | 23.2^[4]^ | 22.2^[4]^ |
| *Oreotragus oreotragus* | 11.3^[4]^ | 13^[4]^ | 10^[4]^ | 0^[4]^ |
| *Oryx dammah* | NA | NA | NA | NA |
| *Oryx gazella* | 178^[4]^ | 166.4^[4]^ | 88.3^[4]^ | 85.8^[4]^ |
| *Ourebia ourebi* | 14.1^[4]^ | 15.1^[4]^ | 13.5^[4]^ | 0^[4]^ |
| *Ovibos moschatus* | NA | NA | NA | NA |
| *Pantholops hodgsonii* | 42.3^[4]^ | 25.8^[4]^ | 61^[4]^ | 0^[4]^ |
| *Pelea capreolus* | 24^[4]^ | 25^[4]^ | 22.5^[4]^ | 0^[4]^ |
| *Philantomba maxwelli* | 6.5^[4]^ | 7.2^[4]^ | 3.5^[4]^ | 2^[4]^ |
| *Philantomba monticola 1* | 4.4^[4]^ | 5.1^[4]^ | 4.5^[4]^ | 3.1^[4]^ |
| *Philantomba monticola 2* | 4.4^[4]^ | 5.1^[4]^ | 4.5^[4]^ | 3.1^[4]^ |
| *Procapra gutturosa* | 31.5^[4]^ | 24^[4]^ | 23^[4]^ | 0^[4]^ |
| *Pseudois nayaur* | NA | NA | NA | NA |
| *Pseudoryx nghetinhensis* | NA | NA | NA | NA |
| *Raphicerus campestris* | 10.9^[4]^ | 11.3^[4]^ | 14.5^[4]^ | 0^[4]^ |
| *Redunca arundinum* | 58.3^[4]^ | 43.2^[4]^ | 37.5^[4]^ | 0^[4]^ |
| *Redunca fulvorufula* | 30.1^[4]^ | 28.5^[4]^ | 26^[4]^ | 0^[4]^ |
| *Rupicapra pyrenaica* | NA | NA | NA | NA |
| *Rupicapra rupicapra* | 40.3^[4]^ | 31.7^[4]^ | 23.2^[4]^ | 21.1^[4]^ |
| *Saiga tatarica* | 42.5^[4]^ | 32.3^[4]^ | 29^[4]^ | 0^[4]^ |
| *Sylvicapra grimmia* | 18.3^[4]^ | 19.6^[4]^ | 9.9^[4]^ | 0^[4]^ |
| *Syncerus caffer* | 642.9^[4]^ | 467.5^[4]^ | 100^[4]^ | 85^[4]^ |
| *Tetracerus quadricornis* | NA | NA | NA | NA |
| *Tragelaphus angasii* | 110.2^[4]^ | 64.4^[4]^ | 65^[4]^ | 0^[4]^ |
| *Tragelaphus derbianus* | 680^[4]^ | 440^[4]^ | 95^[4]^ | 102.5^[4]^ |
| *Tragelaphus eurycerus* | NA | NA | NA | NA |
| *Tragelaphus imberbis* | 95.6^[4]^ | 62.1^[4]^ | 75^[4]^ | 0^[4]^ |
| *Tragelaphus oryx* | 647.3^[4]^ | 415.8^[4]^ | 54^[4]^ | 60.5^[4]^ |
| *Tragelaphus scriptus 1* | 49.7^[4]^ | 31.1^[4]^ | 41^[4]^ | 0^[4]^ |
| *Tragelaphus scriptus 2* | 49.7^[4]^ | 31.1^[4]^ | 41^[4]^ | 0^[4]^ |
| *Tragelaphus scriptus 3* | 49.7^[4]^ | 31.1^[4]^ | 41^[4]^ | 0^[4]^ |
| *Tragelaphus spekii* | 102.3^[4]^ | 60.2^[4]^ | 66^[4]^ | 0^[4]^ |
| *Tragelaphus strepsiceros* | 240.8^[4]^ | 159.2^[4]^ | 120^[4]^ | 0^[4]^ |
| *Giraffa camelopardalis* | 1230.3^[8]^ | 1164.0^[8]^ | 17.5^[8]^ | 14.5 |
| *Antilocapra americana* | 57^[7]^ | 50^[7]^ | 13.3^[20]^ | 4^[7]^ |

**Appendices Literature Cited**

1. Baskin, L., and K. Danell. 2003. Ecology of the ungulates. Springer. Berlin, Germany.

2. Black-Décima, P. 2000. Home range, social structure, and scent marking behavior in brown brocket deer (*Mazama gouazoubira*) in a large enclosure. Mastozool. Neotrop. 7:5-14.

3. Blake, J. E., J. E. Rowell, and J. M. Suttie. 1998. Characteristics of first-antler growth in reindeer and their association with seasonal fluctuations in steroid and insulin-like growth factor 1 levels. Can. J. Zool. 76:2096-2102.

4. Bro-Jørgensen, J. 2007. The intensity of sexual selection predicts weapon size in male bovids. Evolution. 61:1316-1326.

5. Caro, T. M., C. M. Graham, C. J. Stoner, and M. M. Flores. 2003. Correlates of horn and antler shape in bovids and cervids. Behav. Ecol. Sociobiol. 55:32-41.

6. Carranza, J. 1996. Sexual selection for male body mass and the evolution of litter size in mammals. Am. Nat. 148:81-100.

7. Churcher, C. S. 1990. Cranial appendages of Giraffoidea. in Horns, pronghorns, and antlers. G. A. Bubenik and A. B. Bubenik. eds. Springer-Verlag. New York, USA.

8. Dagg, A. I., and J. B. Foster. 1976. The giraffe: Its biology, behavior, and ecology. Van Nostrand Reinhold Company. New York, USA.

9. Duarte, J. M. B., and S. González. eds. 2010. Neotropical cervidology: Biology and medicine of Latin American deer. Funep / IUCN. Jaboticabal, Brazil.

10. Fautley, R., T. Coulson, and V. Savolainen. 2012. A comparative analysis of the factors promoting deer invasion. Biol. Invasions. 14:2271-2281.

11. Geist, V. 1998. Deer of the world: Their evolution, behavior, and ecology. Stackpole Books. Mechanicsburg, USA.

12. {Formatting Citation}Hayssen, V., Ari Van Tienhoven, and Ans Van Tienhoven. 1993. Asdell’s patterns of mammalian reproduction: A compendium of species-specific data. Cornell University Press. Ithaca, USA.

13. Janis, C. M. 1990. Correlation of cranial and dental variables with body size in ungulates and macropodoids. in Body size in mammalian paleobiology. J. Damuth and B. J. MacFaddan eds. Cambridge University Press. Cambridge, U.K.

14. Jones, K. E., J. Bielby, M. Cardillo, S. A. Fritz, J. O’Dell, et al. 2009. PanTHERIA: a species-level database of life history, ecology, and geography of extant and recently extinct mammals. Ecology. 90:2648.

15. Lemaître, J. F., C. Vanpé, F. Plard, and J. M. Gaillard. 2014. The allometry between secondary sexual traits and body size is nonlinear among cervids. Biol. Lett. 10:20130869.

16. Leslie, D. M., Jr. 2009. *Przewalskium albirostre* (Artiodactyla: Cervidae). Mammalian Species. 42:7-18.

17. Loison, A., M. Festa-Bianchet, J. Gaillard, J. T. Jorgenson, and J. Jullien. 1999. Age-specific survival in five populations of ungulates: evidence of senescense. Ecology. 80:2539-2554.

18. Melnycky, N. A., R. B. Weladji, Ø. Holand, and M. Nieminen. 2013. Scaling of antler size in reindeer (*Rangifer tarandus)*: sexual dimorphism and variability in resource allocation. J. Mammal. 94:1371-1379.

19. Mysterud, A., F. J. Pérez-Barbería, and I. J. Gordon. 2001. The effect of season, sex and feeding style on home range area versus body mass scaling in temperate ruminants. Oecologia. 127:30-39.

20. O’Gara, B. W., and J. D. Yoakum. 2004. Pronghorn ecology and management. University Press of Colorado. Boulder, USA.

21. Scott, K. M. 1987. Allometry and habitat-related adaptations in the postcranial skeleton of Cervidae. in Biology and management of the Cervidae. C. M. Wemmer. ed. Smithsonian Institution Press. Washington, DC. USA.

22. Spinage, C. A. 1986. The natural history of antelopes. Facts on File Publications. New York, USA.

23. Weckerly, F. W. 1998. Sexual-size dimorphism: influence of mass and mating systems in most dimorphic mammals. J. Mammal. 79:33-52.
